# An observation-constrained assessment of the climate sensitivity and future trajectories of wetland methane emissions

**DOI:** 10.1126/sciadv.aay4444

**Published:** 2020-04-10

**Authors:** Ernest N. Koffi, Peter Bergamaschi, Romain Alkama, Alessandro Cescatti

**Affiliations:** European Commission Joint Research Centre, 21027 Ispra (VA), Italy.

## Abstract

Wetlands are a major source of methane (CH_4_) and contribute between 30 and 40% to the total CH_4_ emissions. Wetland CH_4_ emissions depend on temperature, water table depth, and both the quantity and quality of organic matter. Global warming will affect these three drivers of methanogenesis, raising questions about the feedbacks between natural methane production and climate change. Until present the large-scale response of wetland CH_4_ emissions to climate has been investigated with land-surface models that have produced contrasting results. Here, we produce a novel global estimate of wetland methane emissions based on atmospheric inverse modeling of CH_4_ fluxes and observed temperature and precipitation. Our data-driven model suggests that by 2100, current emissions may increase by 50% to 80%, which is within the range of 50% and 150% reported in previous studies. This finding highlights the importance of limiting global warming below 2°C to avoid substantial climate feedbacks driven by methane emissions from natural wetlands.

## Teaser

Observation-driven model shows that natural CH_4_ emissions may increase by 50% to 80% in 2100 without action to reduce emissions.

## INTRODUCTION

Methane (CH_4_) is the second most important anthropogenic greenhouse gas after carbon dioxide (CO_2_), with 28 to 34 times the global warming potential of CO_2_ over a 100-year time horizon (*1*, *2*). Atmospheric CH_4_ has significantly increased since the preindustrial times and currently contributes to about 20% of the concentration-based radiative forcing (RF) (*1*). There are both natural and anthropogenic sources of CH_4_ emissions. Wetlands are the largest natural source and contribute between 30 and 40% to the total CH_4_ emissions (*3*–*8*). In parallel, wetlands are important carbon (C) sinks (*9*), since anoxic conditions favor C accumulation by limiting the microbial decomposition of plant residues. Variations under environmental conditions due to anthropogenic disturbances (e.g., draining) or climate change may typically alter the wetlands’ exchange of CO_2_ and CH_4_ with the atmosphere (*10*).

The production of methane in wetlands is due to microbial methanogenesis, a process of anaerobic respiration that occurs in water-saturated soils with limited oxygen availability. Under this environmental condition, methane production depends on temperature, water-table depth, and both the quality and quantity of organic matter (*11*–*14*). Climate change is going to affect these three main drivers of methanogenesis. Current temperature of wetlands over long periods is below the optimum for methanogens [about 37°C (*15*)] in most world regions and will increase worldwide due to global warming. Precipitation is also expected to increase by ∼7% per degree warming (°C^−1^) according to the Clausius-Clapeyron relationship (*16*). Soil-water content will vary locally in response to variations in precipitation patterns, permafrost thawing, and evaporative demands, with wet areas of Earth likely to become even wetter (*17*, *18*), leading in the end to wetland expansion (*19*). Ultimately, substrate availability may also change in response to the observed increase in primary productivity, mainly driven by CO_2_ fertilization (*20*) or by increased bioavailability in carbon-rich soils and permafrost (*21*). Because of the multiple feedbacks between climate and the drivers of CH_4_ emissions, wetlands have the potential to substantially amplify human-induced climate change and are therefore ecosystems of major concern for prediction of future climate trajectories. Recently, using an ensemble of climate projections and a process-based land-surface model (LSM), Zhang *et al.* (*19*) speculate that climate change–induced increases in boreal wetland extent and temperature-driven increases in tropical CH_4_ emissions will dominate future CH_4_ emissions. However, a comprehensive assessment of the role of wetlands in the climate system would also require assessing the CO_2_ sink and the long-term C storage in these ecosystems (*22*), aspects that are beyond the scope of the present paper.

To date, the interplay between climate and wetland CH_4_ emissions has been mostly explored through model-based assessments that have produced rather uncertain and not resolutive findings. For instance, multiple LSMs have been used to predict an increase in wetland CH_4_ emissions by 50 to 150% during the 21st century (*23*–*27*). In addition, LSMs predictions of wetland CH_4_ emissions in future climates typically do not consider the impact of adaptation to the varying environmental conditions. This aspect is of particular relevance since adaptation may substantially dampen the response of the biosphere to the changing climate.

The recent availability of long-term observation-driven estimates of wetland CH_4_ emissions through atmospheric inverse modeling systems opens new opportunities to complement process-based model assessments. An inverse modeling system estimates surface CH_4_ emissions through the use of observed atmospheric CH_4_ concentrations, a transport model, prior estimates of CH_4_ emissions, and estimates of atmospheric CH_4_ removal (primarily by the hydroxyl radical) (*2*, *28*). Using state-of-the-art inversion (INV) estimates and climate data (see “Wetland and wetland datasets” section), we investigated the climate sensitivities of wetland CH_4_ emissions based on the seasonality of both the emissions and climate drivers, with the ultimate goal to produce an observation-driven assessment of future emission trajectories under changing climate conditions in different climate regions. To achieve this objective, we first identify the climate variables that are driving the temporal variability of wetland CH_4_ emissions. Second, we determine the apparent and intrinsic sensitivities of emissions to the selected climate variables. Last, observation-driven methodology is applied to predict future emission scenarios under contrasting hypothesis of full adaptation or no adaptation of wetlands to the projected climate conditions.

## RESULTS

### Dependence of wetland CH_4_ emissions on climate drivers

The temporal variability of wetland CH_4_ emissions has been commonly related to climate drivers at a local scale (*12*–*14*). Here, in contrast, we investigate large-scale climate dependencies simultaneously for five climate zones and on a global scale (see “Wetland and wetland datasets” section). Thus, we analyzed the inverted wetland CH_4_ monthly emissions for the 2000–2012 period produced by the European MACC (Monitoring Atmospheric Composition and Climate) project with the TM5-4DVAR inverse modeling system and observations from the National Oceanic and Atmospheric Administration (NOAA) global cooperative air-sampling network (*2*) (denoted as “MACC_NOAA”). We correlated the inverted fluxes to climate drivers of methanogenesis using various databases (in situ, satellite retrievals, and reanalyses) representing the temperature (air near the surface and soil) and a proxy of water-table depth (precipitation and water anomalies in the ground) of wetland (see “Wetland and wetland datasets” section and fig. S2). We first checked a potential temporal lag between the emissions and climate drivers (i.e., temperature, precipitation, soil water content, and total water column), but our analysis did not indicate any lag at the monthly time scale (details in section S2). In summary, emissions are strongly correlated to air temperature near the surface in all climate zones. On the other hand, in the warmer and arid climate zones, emissions generally show closer correlation with precipitation than temperature, as also reported in previous studies (*29*, *30*). Following the outcome of this exploratory analysis of multiple climate variables, we selected as key climate drivers the mean air temperature and the precipitation from the Climatic Research Unit (CRU) of the University of East Anglia database (*31*).

### Response of wetland CH_4_ emissions to climate variables

To determine the bidimensional response of wetland CH_4_ emissions to temperature *T* and precipitation *P* at global scale, we first factor out the effect of wetland extent and absolute magnitude of emissions observed across pixels. To do so, we normalized the data by dividing the emission observed at each pixel and monthly time step by the mean value of the emissions over the whole period at the same pixel (hereafter referred to as normalized emissions *E*_n_; see “Response of wetland CH_4_ emissions to climate variables” section).

**Fig. 1:**
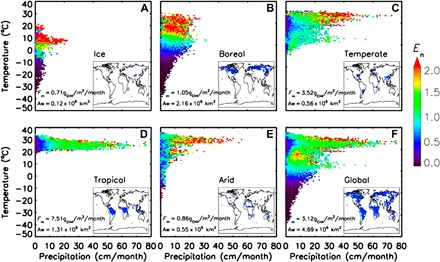
Response of wetland CH_4_ emissions to climate variables. Median value of the normalized emission *E*_n_ as function of the precipitation *P* and the temperature *T* is shown for the five climate zones (**A** to **E**) and at global scale (**F**). Emissions are derived from MACC project over 2000–2012 (MACC_NOAA INV). Precipitation *P* and temperature *T* are from the CRU database. The map of climate zones and the relevant mean value of wetland CH_4_ flux (*F*_m_), together with the wetland area (*A*_w_), are displayed. The sizes of the temperature *T*_c_ and precipitation *P*_c_ bins are set to 1°C and 1 cm/month, respectively.

This analysis characterizes the climate dependence of the assumed global wetland emission of 175 TgCH_4_/year over a total wetland area of 4.69 × 10^6^ km^2^. Results show that the *T* and *P* dependence of wetland CH_4_ emissions is controlled by the background climate with different driving variables in the various climate zones (Fig. 1). Cold regions (ice and boreal, covering about 16% of the total wetland emissions) are mainly sensitive to *T* as suggested by the horizontal stripes in the color pattern of Fig. 1 (A and B). On the contrary, warmer regions [tropics (67%) and arid (3%) of the total emissions] are more sensitive to *P* (vertical stripes in Fig. 1, D and E), while a colimitation by temperature and precipitation is emerging for the temperate climate zone (Fig. 1C). The global response presented in Fig. 1F shows the strong sensitivity of the emissions to *T* in cold climate (below 5°C) and to *P* in warm climates (above 10°C), confirming the findings of previous studies performed at local scale (*12*, *13*, *30*). Similar patterns are found when using alternative INV products and an ensemble of LSMs (see “Wetland and wetland datasets” section and fig. S3), confirming the robustness of the findings.

### Quantification of the sensitivity of wetland CH_4_ emissions to climate variables

Using the response of the emissions to *T* and *P* (Fig. 1), we quantify the sensitivity of the methane emissions to the main climate drivers. In experiments performed with process-oriented models, the sensitivity of a quantity of interest to an independent variable is commonly computed by varying this latter variable, while keeping constant all the other drivers *(**7*). Unfortunately, Earth observations do not allow for a factorial experimental design since all independent variables are varying at the same time. Therefore, the sensitivity has to be characterized by either the total or the partial derivatives of the quantity of interest with respect to the independent variables. While the total derivatives express the apparent sensitivity to a given variable (apparent since it includes also the confounding effects of all the covarying drivers), the partial derivative isolates the intrinsic effect of a single driver, factoring out all other independent variables that are related to the quantity of interest.

Following this logic, the derivatives of the normalized emission *E*_n_ to the climate drivers *T* and *P* have been computed on observational data, considering that the total derivative of *E*_n_ to *T* depends on the partial derivatives of *E*_n_ to both *T* and *P* and by the total derivative of *P* with respect to *T* as follows

$$\frac{{\mathrm{dE}}_{n}}{\mathrm{dT}}=\frac{\partial E_{n}}{\partial T}+\frac{\partial E_{n}}{\partial P}.\frac{\mathrm{dP}}{\mathrm{dT}}$$ (1)

The interplay between the two climate variables *dP*/*dT* has been approximated by the observed slope of the linear relationship between *P* and *T* (∆*P*/∆*T*; see “Quantification of the sensitivity of wetland CH_4_ emissions to temperature and precipitation” section). We derived the apparent (AS_ET_ expressed in percent per degree Celsius) and intrinsic (IS_ET_ expressed in percent per degree Celsius) relative change in emission per degree Celsius of temperature by numerical approximation of the total and partial derivatives of the normalized emission to temperature (see “Quantification of the sensitivity of wetland CH_4_ emissions to temperature and precipitation” section). We used the same method to calculate the sensitivity of emission to precipitation both in terms of apparent effects (AS_EP_ expressed in percent per centimeter per month) and intrinsic effects (IS_EP_ expressed in percent per centimeter per month). To assess the uncertainty of the estimates based on atmospheric inversions, we computed AS_ET_, IS_ET_, AS_EP_, and IS_EP_ on an ensemble of 15 different INV systems. In parallel, we quantify the uncertainty of model predictions analyzing an ensemble of 17 model simulations generated by 11 different LSMs (see “Wetland and wetland datasets” section).

**Fig. 2:**
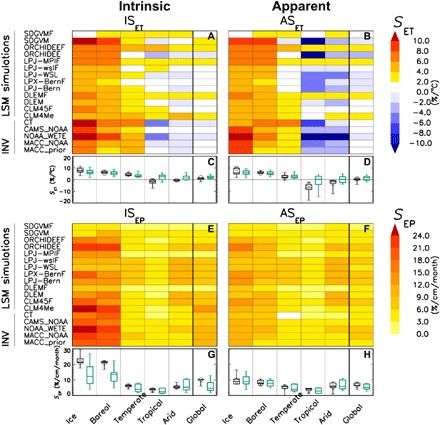
Sensitivity of wetland CH_4_ emissions to temperature *T* and precipitiation *P* for the five climate zones and at global scale. The emissions are derived from both atmospheric inversions (INVs) and LSM simulations. *T* and *P* data are from CRU database. The sensitivity of the emissions to *T* (*S*_ET_; **A**, **B**) and to *P* (*S*_EP_; **E**, **F**) are shown. Results for the intrinsic (IS_ET_ and IS_EP_; **A**, **E**) and for the apparent (AS_ET_ and AS_EP_; **B**, **F**) sensitivities are shown, respectively. For AS_ET_ and AS_EP_, the covariance between climate drivers was estimated using the 3-monthly average slopes between *P* and *T*. The Whisker plots shown at the bottom of each sensitivity matrix graph (**C**, **D**, **G**, **H**) are computed from the ensemble of all the inversions (black) and for all the LSMs (green). The whisker plots show the minimum and maximum S_ET_ or S_EP_ values (bars), and the 25th and 75th percentiles (boxes). The median values are shown by horizontal line in the box. The values of S_ET_ and S_EP_ obtained from the MACC_NOAA INV estimates the reference inversion in this study) are shown by open circles.

Figure 2 (A, B, E, and F) shows the results derived from a subset of these INV and LSM products that are representative of the whole ensemble; results from other INVs and LSMs are reported in fig. S5. Summary statistics computed on all the inversions and all the LSMs are shown in Fig. 2 (C, D, G, and H). An in-depth discussion of these results can be found in section S5. In summary, the analysis shows that a mere increase in the temperature at constant precipitation levels would significantly increase wetland CH_4_ emissions in cold climate regions (ice and boreal), slightly decrease the emissions in the warmer areas (tropical and arid), and have a light positive effect in the temperate climate zone (Fig. 2, A to D). Because of the compensation between climate zones, the global fluxes show slightly positive intrinsic sensitivity (IS_ET_) and negligible apparent sensitivity (AS_ET_) to warming (Fig. 2, A to D). LSMs produce, on average, similar results for cold climate regions but have a high uncertainty not only in the magnitude but also in the sign of the response for the warmer climate zones (Fig. 2, A to D). The higher sensitivity of LSMs to increasing temperatures in the warmer climate zones ultimately results in a slight positive sensitivity to temperature at global scale (about 2%/°C; Fig. 2, C and D). Regarding precipitation, both the INVs and LSMs show similar results, with positive apparent and intrinsic changes in emission in all climate zones and with global sensitivities in the order of 6 to 10%/cm per month (Fig. 2, E to H). It is worth noting that the intrinsic sensitivities to precipitation are larger than the apparent ones, particularly in the colder climate zones.

The difference between apparent and intrinsic sensitivities is driven by the covariation of precipitation and temperature at interannual time scale (section S4). In accordance with Trenberth and Shea (*32*), this short-term covariability gives negative correlations between temperature and precipitation in warmer climate zones and light positive correlations in colder climate zones (fig. S4). The importance of the covariance between temperature and precipitation in determining the response of the biosphere to climate change has been recently stressed also for the CO_2_ ecosystem exchange (*33*).

### Wetland CH_4_ emissions under climate change

Future trends of wetland CH_4_ emissions will depend on the changes in the climate drivers *P* and *T* and of the covariation between them. To investigate the future coevolution of these climate drivers, we used the projection of the ensemble of Coupled Model Intercomparison Project Phase 5 (CMIP5) climate models (*34*) for the four representative concentration pathways (RCPs; see “Wetland and wetland datasets” section). We investigated both the short-term (interannual) covariation between *P* and *T* and the long-term decadal variations (figs. S7 and S8).

The covariation at longer time scale is positive and rather similar across climate scenarios, as it is primarily resulting from the acceleration of the water cycle following warming temperatures, according to the Clausius-Clapeyron relationship. The patterns in the climate domain show the projected trend of wet zones becoming wetter (positive correlation) and dry zone becoming even more arid (negative correlation) (*16*). As previously discussed, the covariation at interannual time scale will depend on the climate zone, with negative values in the warm and water-limited regions. Short-term sensitivities show large intermodel spread but consistent patterns between the different RCPs and across the whole climate domain.

We produced an observation-driven estimate of future CH_4_ emissions from wetlands based on the future long-term dynamic of the climate drivers as derived from CMIP5 and an empirical model based on the responses of the wetland CH_4_ emissions to climate variables similar to that reported in Fig. 1. To do so, we no longer used the concept of climate zones, but instead, we considered regions described by classes of mean temperatures (see “Predicting CH_4_ emissions from global wetlands under climate change” section and fig. S9). This approach leads to a three-dimensional look-up table (LUT) of the emissions as a function of monthly *T* and *P* and annual mean *T*. We used MACC_NOAA INV emission estimates to characterize the response of the wetland CH_4_ emissions to *T* and *P* (from CRU database).

We applied the LUT with two contrasting assumptions: (i) assuming no adaptation and therefore a constant response of emissions to climate drivers or (ii) accounting for a full adaptation of wetland methane emissions to the projected climate (see “Predicting CH_4_ emissions from global wetlands under climate change” section). In addition to accounting for the impact of the changing climate on emission rates, we also considered the variation in emissions due to the potential expansion of wetland areas. Given that the prediction of the wetland extent is not covered by our approach, we used modeled annual wetlands extent from Zhang *et al.* (*19*) to scale up our future predictions. To do so, for each climate model, wetland emissions are multiplied by the ratio of future to current (year 2000) wetland areas, as modeled by Zhang *et al.* (*19*). We stress that using our method with the projected wetland areas at pixel level can introduce inconsistencies; hence, we perform such projections at a climate-zone level (i.e., tropical, arid, temperate, boreal, and ice) as we defined in the “Wetland and wetland datasets” section. We then allocated each pixel to the climate zone covering more than 50% of the grid cell area. Then, we scaled up the total emissions for each climate zone by the projected total wetland areas in this climate zone. Thus, to obtain global estimates, we sum up the emissions from the five climate zones.

Notice that our observation-driven model does not explicitly account for the direct effect of atmospheric CO_2_ concentration on plant primary productivity; hence, the projections miss any variation of the substrate for methanogenis driven by CO_2_ fertilization. Therefore, the projections of future CH_4_ emissions derived with our approach should be interpreted as the direct effect of changing climate conditions, ignoring indirect effects mediated by variations in CO_2_ concentration. Although the understanding of the interplay between atmospheric CO_2_ fertilization and wetland methane emissions is still a challenging research study, simulations of wetland process models (*7*) and empirical CO_2_ enrichment studies (*35*–*37*) generally suggest an increase in wetland methane emissions under CO_2_ fertilization. Consequently, our projections of the future increase in wetland methane emissions might be at lower limit of what could be expected under a scenario of rapidly increasing CO_2_ concentration, such as RCP8.5.

**Fig. 3:**
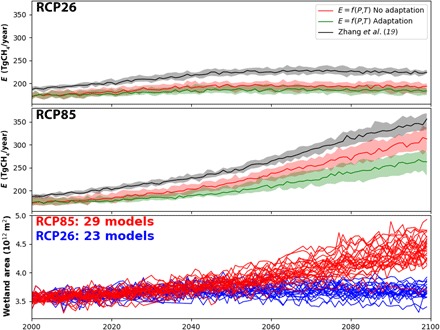
Projected trajectories of wetland CH_4_ emissions. Simulations of wetland CH_4_ emissions using the observation-driven model and climate projections of an ensemble of CMIP5 models for the scenarios RCP2.6 (top) and RCP8.5 (middle). Two versions of the observation-driven model are considered: simulations without any adaptation of the wetland to the new climate (no adaptation; red) and simulations with full adaptation to climate (adaptation; green). Results are compared to those of Zhang *et al.* (*19*) (gray). The observation-driven model estimates are scaled up by the ratio between the projected wetland areas and the computed areas in year 2000 based on Zhang *et al.* (*19*) simulations. Envelopes in the top and middle panels are enclosed by 25th and 75th percentiles of the ensemble predictions. Solid lines are the median values. The bottom graph shows the yearly variations of the computed wetland areas from Zhang *et al.* (*19*) from the ensemble of CMIP5 climate models. The number of models used for each RCP is shown in the bottom graph. The observation-driven method uses for wetland methane emissions the MACC_NOAA INV.

Results for the scenarios RCP8.5 and RCP2.6 are discussed here (Fig. 3), whereas results for the other RCPs are summarized in figs. S10 and S11. When considering only the impact of climate on emission rates for the current wetland area, under scenario RCP8.5, we simulate a significant increase in emissions in 2100, with median values between 140% (assuming full adaptation) and 160% (no adaptation) of the value in 2000. As expected, the simulations under RCP2.6 scenario show a minor increase in the emissions until 2040 and then a relatively stable trend after, reaching about 120% (no adaptation) in 2100 with respect to the emissions in 2000. When the expansion of wetland area is considered, the emissions in 2100 are estimated to be between 150% (adaptation) and 180% (no adaptation) of the value in 2000 using RCP8.5. These results suggest that wetland expansion under climate change might contribute to about 20% of the total wetland emissions in 2100.

**Fig. 4:**
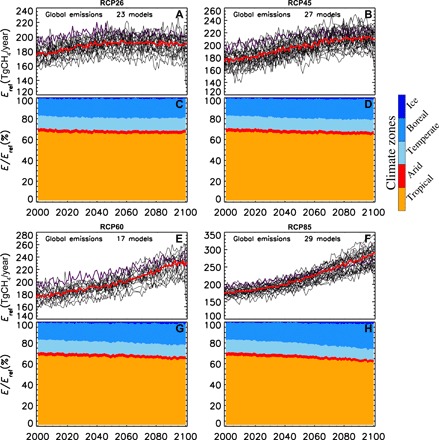
Wetland CH_4_ emissions obtained by combining the observation-driven model, wetland areas from Zhang *et al.* (*19*) and projections of an ensemble of CMIP5 climate models for the four scenarios (RCP 2.6, RCP4.5, RCP6.0, and RCP8.5) based on MACC inversion and assuming no adaptation to climate. Black lines (violet for GISS-E2-R) in the top panel of each scenario (**A**, **B**, **E**, **F**) indicate global emissions computed with individual CMIP5 model outputs; the red line indicates the median of the ensemble. The bottom panels (**C**, **D**, **G**, **H**) show a time series of the percentage contribution from each climate zone.

Our emission estimates that ignore the contribution of new wetland areas are in the lower range of previous findings (*18*–*22*), whereas our estimates corrected by wetland expansion but ignoring adaptation are lower than ones reported by Zhang *et al.* (*19*) and rather similar by the values reported by Shindell *et al.* (*38*) (Fig. 3 and fig. S11). According to (*19*), boreal zones will significantly contribute to these newly developed wetland areas from the thawing of frozen inundated areas under the rising of the temperature during the cold season (December to May). Our regional analysis (Fig. 4) shows that, by the end of this century, the share of tropical wetland emissions is significantly reduced for the scenarios RCP6.0 and RCP8.5 and this is largely compensated by the increased emissions in cold climate zones (boreal and ice). The estimated contribution of the tropical climate zone to the global emissions is currently about 68% and will decrease to 60%, while the contribution of colder regions will almost double (from about 16% at the present time to about 28% at the end of this century). The contributions of the emissions from both the temperate and arid climate zones to the global emissions remain almost stable, with a tendency of a slight decline. Our projected regional wetland methane emissions are compared to those of Shindell *et al.* (*38*) who made projections for the four RCP scenarios with the climate model GISS-E2-R2, which also includes the computation of wetland area dynamics. For this purpose, we used our data-driven method with the projected climate from GISS-E2-R2 to estimate future emissions. We found, on average, that wetland emissions by 2100 for RCP2.6, RCP4.5, RCP6.0, and RCP8.5 are 14, 33, 41, and 90 TgCH_4_/year larger than the emissions in 2005 [i.e., the reference year in (*38*)], respectively (Fig. 4). Overall, projections for the various RCPs are slightly larger, except for RCP8.5, for which our estimate is twice the value reported in (*38*). Both methods suggest a similar increase in wetland emissions in cold regions for the three warmer scenarios, coupled with a reduction of the relative contribution of the emissions from tropical zones. In contrast, we found the largest absolute increase in the emissions in the tropical regions for the scenario RCP8.5, while it is observed for RCP6.0 in (*38*).

The accuracy of the predictions of the observation-driven method with wetland-area correction is limited by the uncertainties in the simulated wetland areas (*7*, *39*). Hence, we suggest further investigations on the future expansion of wetlands based on ensemble of LSMs and climate projections. For this purpose, our method might be coupled to a model of wetland area dynamics as performed in (*38*, *40*).

## DISCUSSION

Our analysis shows that top-down approaches based on inversion modeling may support an observation-driven assessment of methane emissions under past, current, and future climates. Results highlight the relevance of the short- and long-term covariations between the key climate drivers *P* and *T* in projecting the contribution of wetlands to the global CH_4_ budget. The expected long-term positive covariation of *P* and *T*, particularly relevant in the most humid areas of the globe, under the business-as-usual climate scenario (RCP 8.5) will likely lead to an increase in methane emission in the order of 40 to 60% when accounting on for the emission of current wetlands and up to 50 to 80% when considering also wetland expansion. Such an increase in wetland CH_4_ emissions can introduce an additional global mean RF of at least 0.19 W/m^2^ by the end of the 21st century (*19*). Shindell *et al.* (*38*) found similar induced RF but with a substantially lower increase in wetland emissions. The differences between these two computations of the RFs are likely due to the simpler chemistry used in (*19*) as compared to (*38*). On the contrary, under the more ambitious mitigation scenarios (RCP2.6), variation of global wetland emissions should remain within safe boundaries that minimize the risk of large positive feedbacks on the climate system.

## MATERIALS AND METHODS

### Wetland and wetland datasets

#### *Wetland definition*

Wetlands are defined as areas with permanently or temporarily flooded or saturated soils. Wetlands are estimated to cover 6 to 7% of Earth’s surface (*41*) with the largest areas at high latitudes (boreal zones) and the tropics (fig. S1). In this study, we consider wetlands such as floodplains, marshes, and peatlands, and we exclude lakes, rivers, and coastal wetlands.

#### *Domain of study and definition of climates zones*

We examine the sensitivity of wetland methane (CH_4_) emissions to climate variables at global scale using five climate zones based on Köppen-Geiger classification. We use the latest digital Köppen-Geiger World map based on datasets from the CRU (*31*) at the University of East Anglia and the Global Precipitation Climatology Centre [GPCC; (*42*)] at the German Weather Service (*43*). The map contains 31 climate zones on a regular 0.5° latitude/longitude grid for the period 1951 to 2000. We merged the 31 climate zones into five major zones (ice, boreal, temperate, tropical, and arid; fig. S1), as defined in the classification system of (*43*).

#### *Wetland CH_4_ emissions: Atmospheric inversion-based estimates and LSM simulations*

To quantify the sensitivity of wetland CH_4_ emissions to climate variables, we consider emissions estimated from both atmospheric inversions and LSMs. Inverse models estimate surface CH_4_ emissions through the use of observed atmospheric CH_4_ concentrations, an atmospheric transport model, prior fluxes of CH_4_ emissions, and estimates of atmospheric CH_4_ removal (primarily by the hydroxyl radical). We use the wetland CH_4_ emissions estimated from the TM5-4DVAR inverse modeling system (*2*, *44*) as main reference in this study. The TM5-4DVAR system is built around the global chemistry transport model TM5 (*45*) and uses 4DVAR variational technique to optimize emissions of individual grid cells, using an adjoint model that iteratively minimizes the cost function using a quasi-Newton (*46*) algorithm. The applied inversion optimizes four groups of methane emission sources: wetlands, rice, biomass burning, and all remaining source categories using prior information about these sources as described in (*2*). The four emission groups are separated during the optimization process based on their different spatial distributions and seasonal variations (*2*). For wetlands, the prior fluxes are from the wetland inventory of Jed Kaplan [described in (*2*)]. The defined wetlands cover an area of 6.2 10^6^ km^2^ globally. We use the inverted wetland emissions over the 2000–2012 period, generated during the European MACC (inversion “v10-S1NOAA_ra”) project (*2*). Furthermore, we use additional TM5-4DVAR inversions using either different prior wetland fluxes or different CH_4_ observational datasets in the assimilation process (in situ measurements and satellite observations). We analyze 13 different inversions based on the European Commission Joint Research Centre (JRC) TM5-4DVAR system (table S1). In addition, we include the CH_4_ inversion from the European Copernicus Atmosphere Monitoring Service (CAMS; inversion “v16r1”) (*47*) in our analysis. The CAMS inversion system is largely based on the TM5-4DVAR system developed by JRC during the MACC project but includes some updates and slightly different settings (*47*, *48*). Furthermore, we examine the wetland CH_4_ emissions from the NOAA CarbonTracker-CH_4_ (CT-CH_4_) inverse system (*49*). For the latter, we consider wetland CH_4_ emissions as the difference between the natural emissions and the ocean emissions. However, the natural emissions in CT-CH_4_ include emissions from termites and wild ruminants, but these sources are assumed to be much smaller (~25 TgCH_4_/year) than wetlands. To limit the impact of these additional nonwetland natural emissions for this study, we apply the mask of the spatial distribution of wetland areas from Kaplan (*50*). In total, we use 15 different inversion estimates (table S1).

We also test the wetland CH_4_ emissions simulated by an ensemble of LSMs, over the 1999–2004 period from the WETCHIMP [Wetland and Wetland CH_4_ Inter-comparison of Models; (*7*)] project and recent LSM simulations, over 2000–2012, reported in (*6*). The LSMs simulated both wetland CH_4_ emissions and wetland dynamic areas. Within WETCHIMP, each model used its own spatial and temporal distributions of wetland areas (varying between 7 × 10^6^ and 27 × 10^6^ km^2^ globally), and large uncertainties between the models’ estimates were found. On the other hand, Poulter *et al.* (*6*) used as input the same spatial and temporal distributions of wetland areas based on remote sensing and inventory data, with a maximum global area of 10.5 × 10^6^ km^2^. In this study, we use 17 different LSM datasets (table S2).

#### *Climate data for current period: In situ measurements, satellite retrievals, and reanalyses*

We analyze various data sources relevant for the drivers of wetland CH_4_ emissions, including water-table depth, precipitation, soil-water content, and temperature (air and soil) from observations (in situ and satellite) and reanalyses (table S3). To characterize the temperature in wetlands, the monthly mean temperature, monthly average daily minimum, and monthly daily maximum air temperatures near the surface from CRU are used (*31*). Furthermore, the air temperature at 2 m and soil temperatures at four level depths from the European Centre for Medium-Range Weather Forecasts (ECMWF) ERA (ECMWF Re-analysis)–Interim reanalyses (*51*) are considered. For the water table, as proxy, the two gridded precipitation datasets based entirely on gauge information developed by CRU (*31*) and GPCC (*52*) are used. We also use the Global Precipitation Climatology Project precipitation (GPCP) based on sequential combination of microwave, infrared, and gauge data (*53*). In addition, the soil-water content at four level depths from the ERA-Interim reanalyses (*51*) is analyzed. Furthermore, we test the monthly land mass grids that contain terrestrial water storage anomalies (in aquifers, river basins, etc.) from twin satellites of the Gravity Recovery and Climate Experiment (GRACE) (*54*). Last, we examine the Land Surface Water Index (LSWI) of MODIS (Moderate Resolution Imaging Spectrometer) (*55*) derived from the shortwave infrared and the near-infrared regions of the electromagnetic spectrum. LSWI is known to be sensitive to the total amount of liquid water in vegetation and its soil background (*56*). In summary, we test eight variables linked to the temperature and nine related to the water-table depth (table S3).

#### *Climate data for the 21st century: CMIP5 projections*

To predict the wetland CH_4_ emissions in future climate with our empirical model based on the response function of the emissions to climate variables (see “Predicting CH_4_ emissions from global wetlands under climate change” section), we make use of the temperature of air near the surface and precipitation simulated by an ensemble of Earth system models within the CMIP5 (*34*). These models provided historical runs (1950–2005) using observations of greenhouse gases concentrations, aerosols, and land-use changes and future runs (2006–2100) under the four RCPs (RCP2.6, RCP4.5, RCP6.0, and RCP8.5), being the greenhouse gas concentration trajectories adopted by the Intergovernmental Panel on Climate Change for its fifth assessment report (*57*). The RCP is defined on the basis of the RF for the predicted climate in 2100. As an example, the RCP2.6 peaks in RF at about 3 W/m^2^ around 2040 to reach 2.6 W/m^2^ in 2100, while RCP8.5, the worst scenario for climate change, shows a rising of RF to reach 8.5 W/m^2^ in 2100. We use 110 model outputs relevant for the four RCPs (table S4).

#### *Data processing*

All datasets (emissions and climate data) have been regridded at 6° (longitude) by 4° (latitude) spatial and monthly resolution, which is the spatial and temporal resolutions of the reference emission product (MACC inversion “MACC_NOAA”). We consider only the pixels with wetland CH_4_ emissions and with the dominant climate zone representing an area greater than 50% of the grid cell area. Note that for the empirical model simulations of the future climate, we no longer use the concept of climate zones, but we consider regions described by classes of mean temperatures (see “Predicting CH_4_ emissions from global wetlands under climate change” section and fig. S9). We factor out the effect of wetland extent and absolute magnitude of the emissions observed across the pixels. Thus, for each pixel, we normalize the monthly emissions by their mean values computed over the common period of emission and climate data (hereafter *E*_n_). This also removes partly the long-term variations of the carbon substrate.

### Response of wetland CH_4_ emissions to climate variables

We compile a LUT of the responses of wetland CH_4_ emissions to both the temperature *T* and the precipitation *P* individually and simultaneously for the five climate zones and globally. For this analysis, we binned the emission data for each climate zone in classes of temperature centered on *T*_c_ (bin size ∆*T*_c_) and precipitation centered on *P*_c_ (bin size ∆*P*_c_). For each grid cell of a processed region (climate zone or global scale), we go through the monthly series of both the normalized emissions *E*_n_ and climate data (*T* and *P*) and attribute *E*_n_ to the relevant climate bins (*T*_c_ and *P*_c_). We do so for all the grid cells composing the processed region. For each climate bin, we compute both the mean and the median values of *E*_n_. In the following analysis, we choose the median value of *E*_n_ as it is more appropriate for skewed distribution. Examples of LUT of the responses of *E*_n_ to both *T* and *P* are given in Fig. 1 and fig. S3.

### Quantification of the sensitivity of wetland CH_4_ emissions to temperature and precipitation

Using the Eq. 1, the apparent sensitivity of the emissions to temperature (AS_ET_) is given by the total derivative *dE*_n_/*dT*, which depends on the partial derivatives of *E*_n_ to *T* and *E*_n_ to *P*, and the covariation between *P* and *T* (total derivative of *P* with respect to *T*). The inherent effect of a temperature variation on emissions at constant level of precipitation is expressed by the intrinsic sensitivity (IS_ET_) computed as the partial derivatives of *E*_n_ with respect to *T*.

A numerical method to estimate the apparent and intrinsic sensitivities of emissions to climate drivers in observational datasets is to quantify the response of the system to the drivers (with a LUT or parameterized equations) and apply this response to a dataset of perturbed drivers. Following this logic, to compute the apparent sensitivity *dE*_n_/*dT*, we first perturb the time series of climate records *T* and *P* at each grid cell as follows

$$\left\{ \begin{array}{c} T_{p}=T\mp \delta T\\ P_{p}=P\mp \delta T.\frac{\Delta P}{\Delta T} \end{array}\right.$$ (2)

where *T*_p_ and *P*_b_ are the perturbed *T* and *P*, respectively, and δ*T* is the amplitude of the temperature perturbation. The term ∆*P*/∆*T* is approximated by the slope of the linear relationship between observed *P* and *T*, as described in section S4. To account for the seasonal variation, we computed the term ∆*P*/∆*T* for individual season.

Using a LUT of normalized emission *E*_n_ for classes of *T* and *P* (e.g., Fig. 1), we compute the cumulative annual emissions over the entire processed region (i.e., a given climate zone or at global scale) for the perturbed datasets Cf_−δ*T*_(*P*,*T*) and Cf_δ*T*_(*P*,*T*). The sensitivity of *E*_n_ to temperature is ultimately computed as the ratio between the variation in cumulated emissions induced by the climate perturbation, divided by the size of the perturbation (2δ*T*), and normalized by the cumulative annual emissions obtained from the climate records of *T* and *P* [Cf_0_(*P*,*T*)]

$${\text{AS}}_{\text{ET}}(\%/{}^{\circ}C)=(\frac{{\text{Cf}}_{\delta T}-{\text{Cf}}_{-\delta T}}{2\delta T}).\frac{100}{{\text{Cf}}_{0}}$$ (3)

Following the same method, we derive the intrinsic sensitivity of *E*_n_ to *T* (IS_ET_ expressed in percent per degree Celsius) by perturbing *T* as in Eq. 2, maintaining *P* unchanged, and then applying Eq. 3 to emissions computed with the perturbed climate. In this way, we isolate the effect of a single variable from the covariate, mimicking the potential outcome of a factorial model experiment.

The same approach is used for the computation of the apparent (AS_EP_) and intrinsic (IS_EP_) sensitivities of the wetland CH_4_ emissions to precipitation. Sensitivities to precipitation are expressed in percent per centimeter per month. We performed a sensitivity test to find the proper size of the perturbations δ*T* and δ*P* and found that δ*T* less than or equal to 1°C and δ*P* less than or equal to 2 cm/month are reasonable for the analyses, since IS_ET_, AS_ET_, IS_EP_, and AS_EP_ are rather constant for this range of perturbations.

### Predicting CH_4_ emissions from global wetlands under climate change

We investigate the response of wetland CH_4_ emissions under different climate scenarios and two opposite and extreme assumptions concerning the capacity of wetland to adapt to the new environmental conditions. Thus, we parameterize the response of the wetland CH_4_ emissions to *T* and *P* for different classes of mean temperatures, as performed for the climate zones (Fig. 1 and fig. S3). This corresponds to a three-dimensional LUT of the emissions as a function of monthly *T* and *P* and annual mean *T* (fig. S9).

In the first case, we assume that wetland will not adapt to climate warming and, therefore, the response of emission to climate in a given pixel will not change with the variation of the background climate. In the second case, we assume that wetland will have a perfect capacity to adapt to the new temperature regime and, therefore, the emission responses will fully adjust to the new climate, becoming equal to those originally observed for that climate conditions.

These two cases represent two extreme conditions in terms of future ecosystem responses. The real response will likely be in between these two scenarios that should therefore frame the potential future emissions.

To derive future emissions from observations under the two cases of adaptation, we proceed as follows:

1) We compute the mean annual temperature for each grid cell over the initial 10 years (here, 2000–2010 period). Grid cells are classified in classes of mean temperature *T*_mC_ (with bin size ∆*T*_mC_ set to 2°C). *T*_mC_ ranges from −40° to 40°C with 1°C step.

2) For the pixel falling in a given *T*_mC_, the response of the normalized emission *E*_n_ to climate (monthly *T* and *P*) is characterized with a LUT, as shown in fig. S9. As climate data, precipitation and temperature from the CRU dataset have been used. As emission data, we use MACC_NOAA INV estimates for the period 2000–2012, which is representative of the mean sensitivities of the emissions to climate variables in the ensemble of the inversion products (Fig. 2).

3) Under the assumption of no adaptation, the class of *T*_mC_ of each grid cell and, therefore, the LUT defining its response to climate remain unchanged for the entire course of the simulation, and it corresponds to that of the period 2000–2010. On the contrary, under the assumption of full adaptation, the *T*_mC_ is dynamically recalculated over the 10-year interval centered on the simulation year, and the LUT changes accordingly.

4) The emissions under a given climate scenario are computed as a function of the predicted climate scenario, and the response function related to the *T*_mC_ of the grid cell is as follows

$$E_{i}=f_{\text{TmC}}(P_{c},T_{c})*f_{\text{pm}}$$ (4)

where *f*_pm_ is the mean value of the wetland CH_4_ emission in the pixel *i* over the whole period of observations. *f*_TmC_(*P*_c_,*T*_c_) stands for the median value of normalized emission *E*_n_ in class (*P*_c_,*T*_c_) for the relevant *T*_mC_ class. In case the LUT does not have observations in the assigned climate class (*T*_c_,*P*_c_), the weighted *E*_n_ values of the nearby classes are used. As weight, we used the inverse of their Euclidian distance to the position (*T*_c_,*P*_c_) with power four to give more importance to the closest data points (*58*).

5) Emissions according to Eq. 4 are computed using the predicted temperature and precipitation over 2000–2100 from an ensemble of the CMIP5 models (table S4).

6) Global emissions are lastly computed as the sum of the emissions *E*_i_ computed for each grid cell with wetland.

## Supplementary Material

aay4444_SM.pdf

## SUPPLEMENTARY MATERIALS

Supplementary material for this article is available at http://advances.sciencemag.org/cgi/content/full/6/15/eaay4444/DC1
