# Supplementary material for: An observation-constrained assessment of the climate sensitivity and future trajectories of wetland methane emissions
Source: Sci Adv. 2020 Apr 10;6(15):eaay4444. doi: 10.1126/sciadv.aay4444 (PMC7148105; doi:10.1126/sciadv.aay4444)
Supplement: aay4444_SM.pdf [file aay4444_SM.pdf]

Science Advances
AAAS

advances.sciencemag.org/cgi/content/full/6/15/eaay4444/DC1

Supplementary Materials for

An observation-constrained assessment of the climate sensitivity and future trajectories of wetland methane emissions

Ernest N. Koffi*, Peter Bergamaschi, Romain Alkama, Alessandro Cescatti

*Corresponding author. Email: ernest.koffi@ec.europa.eu

Published 10 April 2020, *Sci. Adv.* **6**, eaay4444 (2020)
DOI: 10.1126/sciadv.aay4444

This PDF file includes:

Sections S1 to S7
Figs. S1 to S11
Tables S1 to S4
References

S1: Domain of study

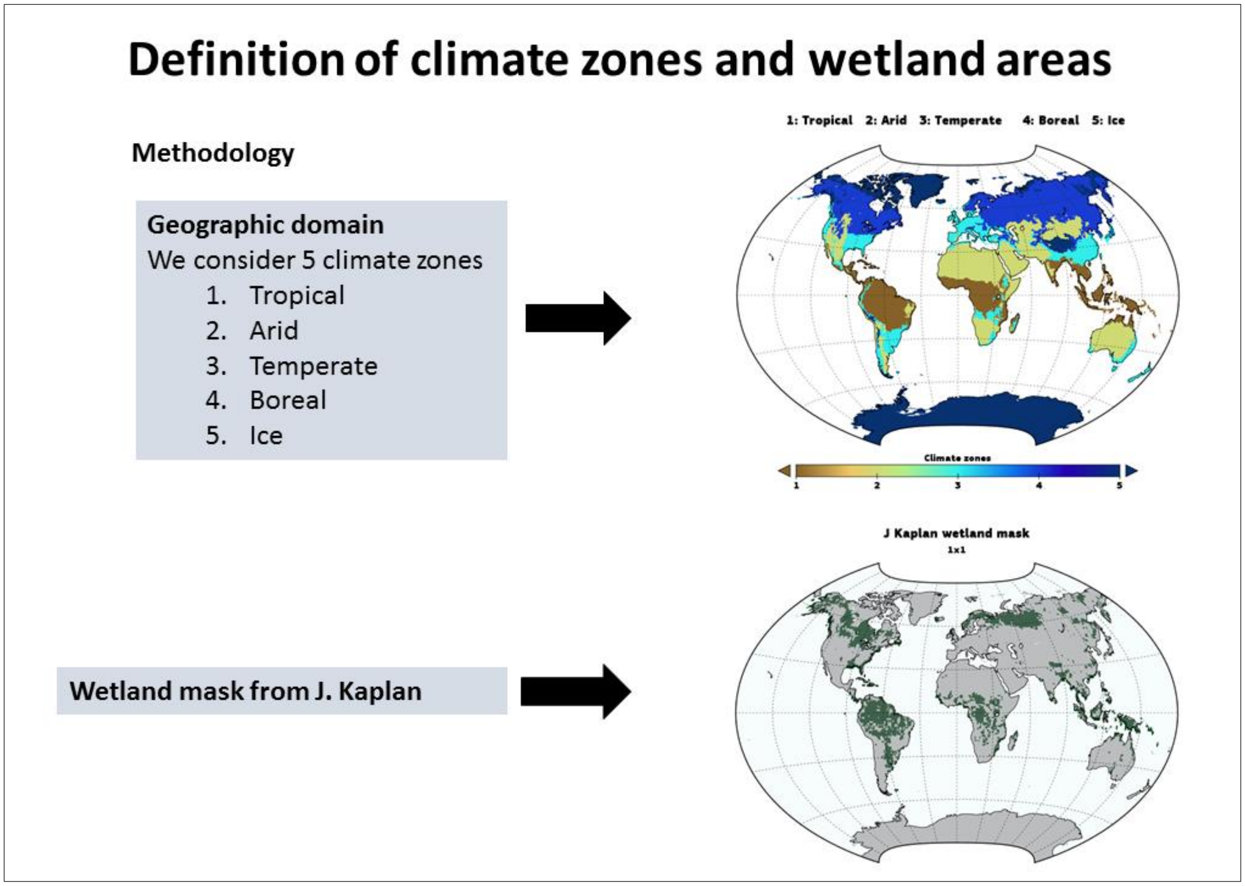

Figure S1: Domain of study with the five climate zones (top). The wetland mask from Kaplan (50) is shown (bottom).

S2: Dependence of wetland CH_4 emissions on climate drivers

The temporal variability of wetland CH_4 emissions has been commonly related to climate drivers at local scale (12-14). On the contrary, here we investigate large scale climate dependencies for five climate zones and at global scale (**Methods 1**). For this scope we analyzed the inverted wetland CH_4 monthly emissions for the 2000-2012 period produced by the European MACC (Monitoring Atmospheric Composition and Climate project) with the TM5-4DVAR inverse modelling system and observations from the National Oceanic and Atmospheric Administration (NOAA) global cooperative air sampling network (2) (denoted "MACC_NOAA"). We first checked a potential temporal lag between the emissions and climate drivers, but our analysis did not indicate any lag at the monthly time scale. Secondly, we computed for each climate zone and at global scale the mean seasonal cycles of both the inverted wetland CH_4 emissions and the climate drivers, and then calculated the linear correlation coefficients between them (**Fig. S2**). As expected, emissions are positively and significantly correlated to soil temperature (top three layers, ECMWFstl1- ECMWFstl3) derived from ECWMF re-analyses (51) in all climate zones except the Arid. Moreover, emissions are also strongly correlated to air temperature near the surface (monthly minimum (CRUtmn) / mean (CRUtmp) / maximum (CRUtmx)) derived from the CRU database (31). Strong positive correlations are observed in all climate zones between the emissions and i) total precipitation, regardless the data source (CRU_P (31), GPCC (42), and GPCP (53)) and ii) the water content in the deepest soil layer (ECMWFstl4: a depth of between 1.0 m and 2.89 m) in regions with high precipitation rates when using ECMWF re-analyses. Generally, in the warmer and arid climate zones, emissions show closer correlation with precipitation than temperature, as also reported in previous studies (29, 30). Satellite retrievals of anomalies in the total water column (GRACE; (54)) and of water content of leaves (MODIS Leaf Surface Water Index (LSWI); (56)) weakly correlate or even anti-correlate to wetland CH_4 emissions. Concerning GRACE, our results are in line with those reported in (30), which emphasize the relatively good correlation between emissions and GRACE retrievals in the tropics and a weak correlation or anti-correlation at northern latitudes. The results at high latitudes can be explained by the positive correlation between snow depth and observed total water column in this region, which can go in the opposite direction with CH_4 emissions. Our results also indicate the limited capability of LSWI to characterize the water status of the ecosystem, since this spectral index gives information only on the water content near the surface. Given the outcome of this exploratory analysis of multiple climate variables, for the following analyses we selected as key climate drivers the mean air temperature (CRUtmp) and the precipitation (CRU_P) from the CRU database.

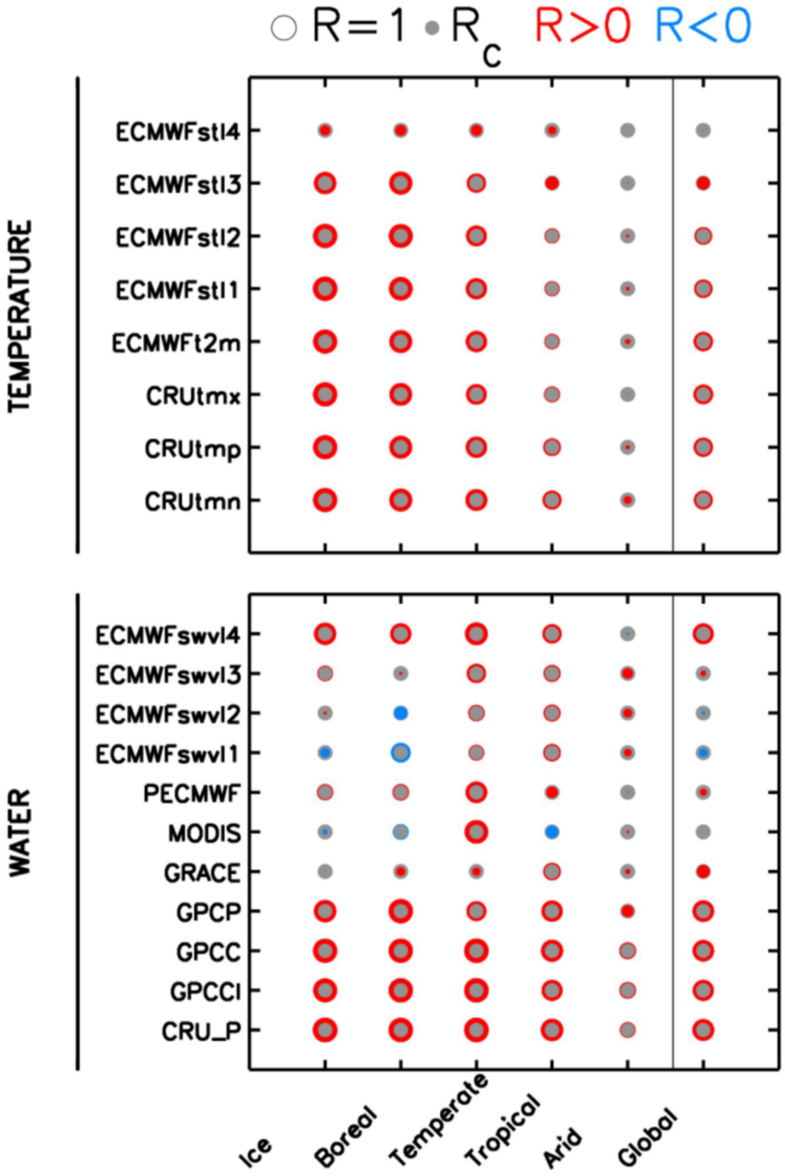

Figure S2: Correlation between mean seasonal wetland CH_4 emissions and mean seasonal climate data. Linear correlation coefficients (R) between inverted CH_4 emissions from MACC project (MACC_NOAA) and each of the selected climate variables for five climate zones and at global scale representing the temperature and water table are shown. R_C (grey dot) equal to 0.58 is the value of R above which the linear relationship (12 pairs of data) is significant at 95% confidence level. The different data sources are described in Methods 1.

S3: Response of wetland CH_4 emissions to climate variables

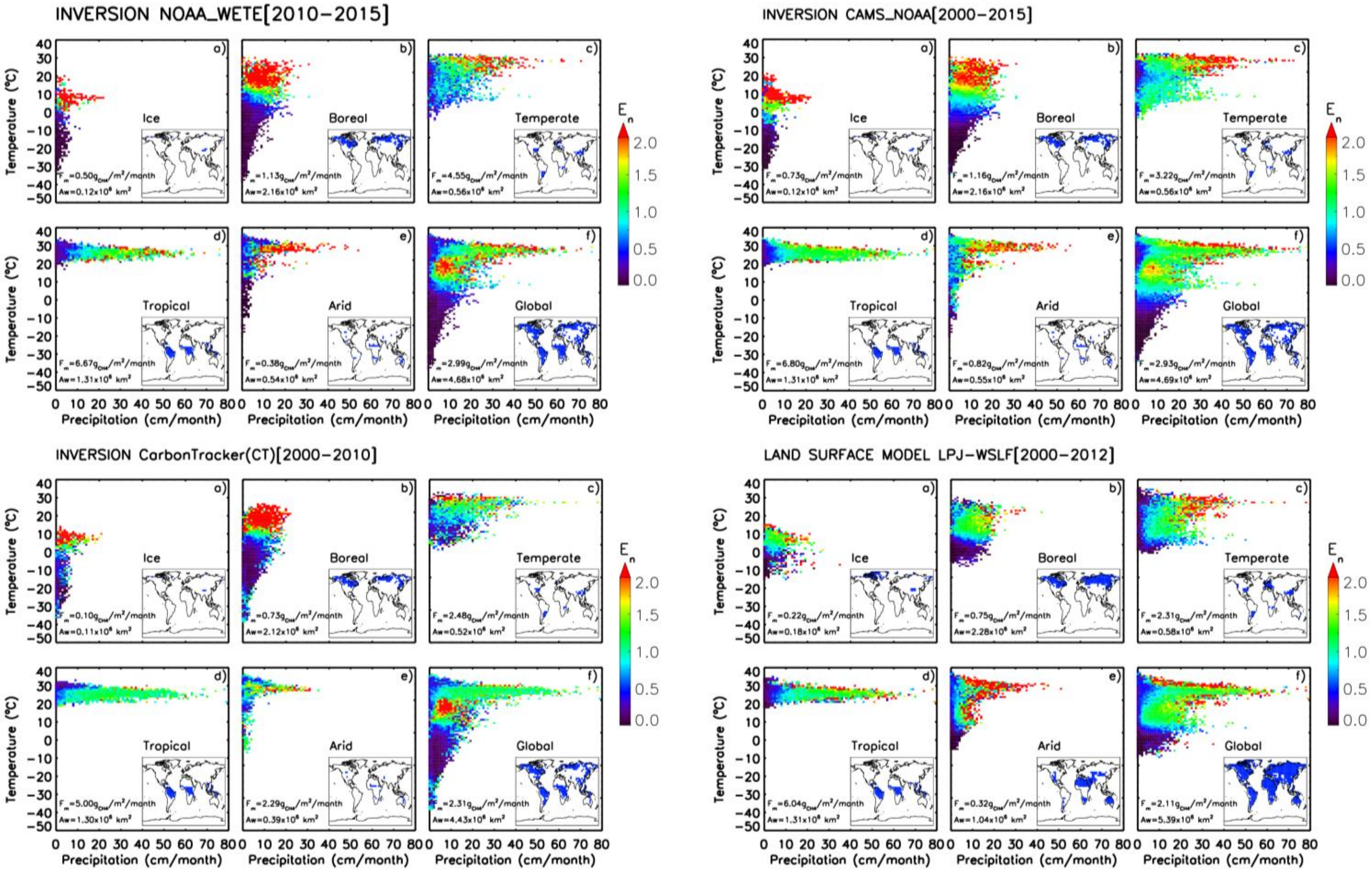

Figure S3: Response functions of wetland CH_4 emissions to precipitation P and temperature T. Median values of the normalized emission E_n as function of P and T are shown for the five climate zones and at global scale. The acronyms and period of the emissions are indicated on the top of each graph. P and T are from the CRU database. The map of climate zones and the relevant mean values of wetland CH_4 fluxes together with the wetland areas are displayed. The bin sizes of the classes T_c and P_c are set to 1°C and 1 cm/month, respectively. Only pixels showing wetland emissions are considered.

S4: Quantification of dP/dT

To quantify the apparent sensitivity of the emissions to either the temperature (AS_{ET}) or the precipitation (AS_{EP}), we need to estimate dP/dT (**Methods 3**). dP/dT is approximated by the slope (α) of the linear relationship between P and T. We compute α at each pixel and for each month of the year by using different P and T data sets as follows:

- We use the data for each month over the period of study
- We use the data for each season. The computed α is attributed to each month of the processed season. We do that for several types of season (season at 2 months (2M), 3M, 4M, 6M, 12M)

As examples, the spatial distributions of α computed at monthly and three seasonal scales (2M, 3M, 4M) are investigated. The monthly slopes are theoretically the more accurate, but their real accuracy is limited by the small data sample (here at most the number of years of the observations), hence monthly α can show large uncertainty. At the same time, the use of data from intervals of several months (which allows a large data sample), gives α that average seasonal variabilities of both P and T. As a compromise, we derived α at 3 months (3M) resolution, as shown in **Fig. S4**. Results show that strong negative slopes dominate in the warmer climate zones, while in the colder zones the slopes are dependent on the seasons, with light positive slopes in winter, spring, and autumn and light negative slopes in summer.

We investigate the sensitivity of wetland CH_4 emissions to temperature and precipitation to the computation method of α. We find that AS_{ET} and AS_{EP} obtained by α computed by using seasons at 2 and 4 months (2M, 4M) do not differ much to the values of AS_{ET} and AS_{EP} using season at 3 months (not shown).

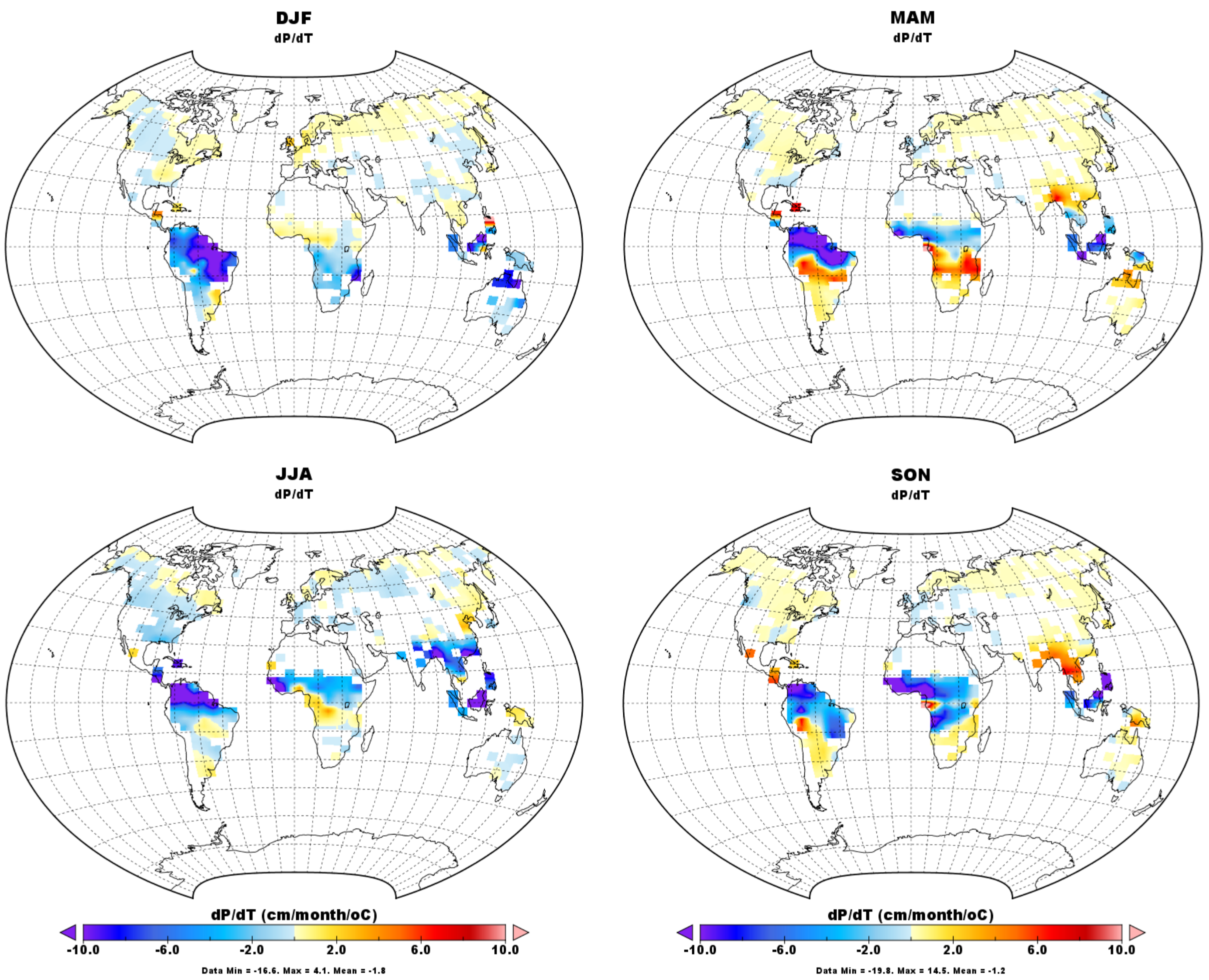

Figure S4: Relationship between the precipitation P and the temperature T (dP/dT). The spatial distrubtions of the slopes derived from the linear relatiosnhip between P and T for each of the 4 seasons of the year over the 2000-2012 period are shown. P and T are from CRU data base. DJF (December, January, February), MAM (March, April, May), JJA (June, July, August), SON (September, October, November)

S5: Quantification of the sensitivity of wetland CH_4 emissions to climate variables

We quantify both the apparent and intrinsic sensitivities of the emissions to temperature (AS_{ET} and IS_{ET}) and precipitation (AS_{EP}, IS_{EP}) for each climate zone and at global scale using the response functions of the emissions to T and P as described in **Methods 3.** AS_{ET}, IS_{ET}, AS_{EP}, and IS_{EP} are computed using an ensemble of 15 different estimates of global emissions from inversion systems (INV) and an ensemble of 17 outputs of land surface models (LSM) (**Methods 1**). **Fig. 2abef** shows the results derived from a subset of these INV and LSM products that are representative of the whole ensemble; results from other inversions and land surface models are shown in **Fig. S5**. Summary statistics computed on all the inversions and all the LSMs are displayed in **Fig. 2cdgh**.

Looking at the intrinsic temperature sensitivity (IS_{ET}; **Fig. 2a**), large positive values that can reach up to 10%/°C (even above in some cases), are found for the colder climate zones (Ice and Boreal) from almost all the processed data sets (excluding SDGVMF model). On the contrary, zero or even small negative values of IS_{ET} are obtained for all the inversions and most of the land surface models for Tropical and Arid climate zones, while small positive values are found for the Temperate climate zone. When the covariance between precipitation and temperature is not factored out, the apparent temperature sensitivities (AS_{ET}; **Fig. 2b**) for cold climate zones do not differ significantly from the intrinsic sensitivities, whereas they decrease substantially for the other climate zones, being strongly negative for Tropical and Arid regions for most of the inversion and model-based emission estimates, with the exception of some land surface models that show positive sensitivities (ORCHIDEEF, DLEMF, CLM4Me, SDGVMF; **Figs. 2 and S5**). Because the Tropical climate zone dominates wetland methane emissions, at global scale both IS_{ET} and AS_{ET} are close to zero or even slightly negative for the inversion products, while positive AS_{ET} values are also found for some land surface models (**Figs. 2ab and S5**). It is worth mentioning that IS_{ET} and AS_{ET} depend on the temperature metric used in the analysis (**Fig. S6**). Thus, the sensitivity of the emissions to the monthly average of daily minimum temperature gives larger positive values of IS_{ET} and AS_{ET} for almost all the emission products (inversions and LSM simulations) regardless the climate zones. Conversely, the negative IS_{ET} and AS_{ET} values obtained from the Tropical and Arid climate zones are amplified when considering the monthly average daily maximum temperature. Concerning sensitivity to precipitation, all values are consistently positive for all the emission estimates (inversions and LSM simulations), with larger values of the intrinsic sensitivities than the apparent ones, in particular in the colder climate zones (**Fig. 2efgh**).

The difference between apparent and intrinsic sensitivities is driven by the co-variation of precipitation and temperature at inter-annual time scale. In accordance with Trenberth and Shea (32), this short-term co-variability gives negative correlations between precipitation and temperature in warmer climate zones and light positive correlations in colder climate zones, as shown in **Fig. S4**. The negative correlations in warmer zones are mainly due to dry conditions on land, which favor more sunshine and less evaporative cooling, while in wet summers temperatures are mitigated by larger cloud cover, reduced solar irradiance and enhanced evaporative cooling. For colder climate zones, two mechanisms compete: in winter, colder conditions limit the water holding capacity of the atmosphere with little precipitation. In contrast, during warmer seasons the mechanism is similar to the one of warmer climate zones. The importance of the covariance between temperature and precipitation in determining the response

of the biosphere to climate change has been recently stressed also for the CO_2 ecosystem exchange (33).

The intrinsic sensitivities (IS_{ET} and IS_{EP}) compared with the changes in emissions due to both temperature and precipitation as derived from WETCHIMP project (7) give contrasting results. As in this study, the LSMs in WETCHIMP give positive emission changes to increased precipitation everywhere. However, for the emission changes to temperature, our sensitivity estimates are partly different from the WETCHIMP analysis. In the colder climate zones, our method gives positive emission sensitivities to temperature for all the LSMs, while several LSMs give negative changes in WETCHIMP. Moreover, in warmer climate zones, the WETCHIMP found large negative emission changes to temperature for almost of the LSMs, while our method shows zero or slight positive IS_{ET} values (**Fig. S5**). The differences can partly be explained by the different methodology used in WETCHIMP to derive the sensitivities (7). As an example, to calculate the change in temperature, the authors assumed that we will experience a warming of 3.5 $^{\circ}$C in 2100. Thus, they added uniformly this temperature increment to the present temperature at each pixel. Then, the change in emissions due to both temperature and wetland areas is obtained by the difference between the simulated emissions in 2100 and the simulations of the present climate.

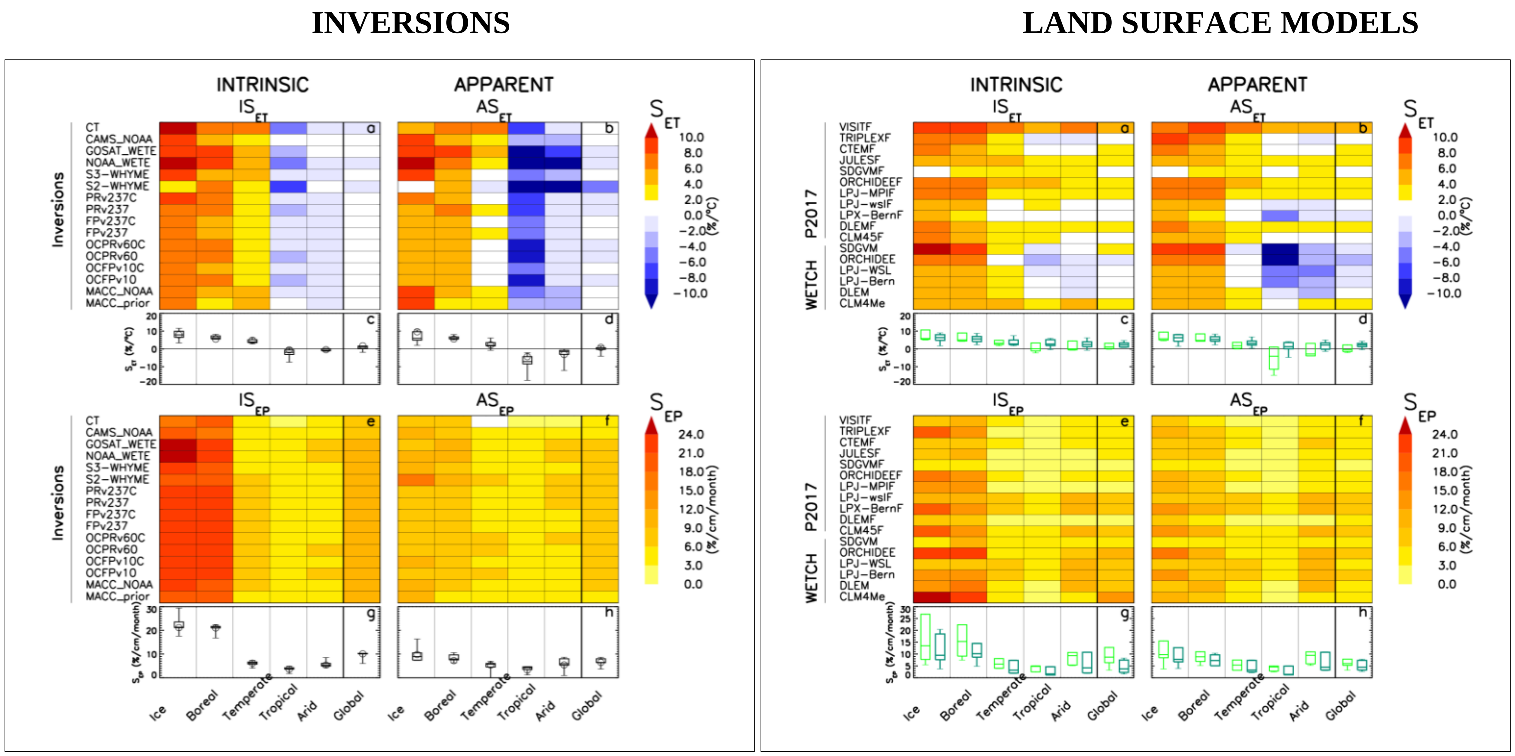

Figure S5: Sensitivity of wetland CH_4 emissions to climate variables for the five climate zones and at global scale. The left panel shows the sensitivities of emissions estimated from the inversions (CarbonTracker-CH4 (CT) and TM5-4DVAR (all the other emissions) inverse modelling systems). The right panel shows the sensitivities of the wetland emissions simulated by land surface models within WETCHIMP (WETCH; Melton, *et al.* (7)) project and those reported in Poulter, *et al.* (6) (P2017). T (monthly mean of daily value) and monthly P data are from the CRU database. For each panel, the sensitivity of the emissions to T S_{ET} (top) and to P S_{EP} (bottom) are shown. Results for the intrinsic (IS_{ET} and IS_{EP} computed assuming no co-variability between P and T; left) and for the apparent (AS_{ET} and AS_{EP} with co-variability between P and T; right) are shown, respectively. The slopes between P and T are computed using a season at 3 months. The Whisker plots shown on the bottom of each sensitivity matrix graph are derived from all emission estimates (all inversions and all land surface models, respectively). The whisker plots show the minimum and maximum S_{ET} or S_{EP} values (bars), and the 25% and 75 % percentiles (boxes). The median values are shown by horizontal line in the box. On the left, the values of S_{ET} and S_{EP} obtained from the MACC inversion estimates (reference inversion in this study: MACC_NOAA) are shown by open circles. On the right, the Whisker plots for S_{ET} and S_{EP} from WETCHIMP (light green) and for P2017 (green) are shown in the bottom of each matrix graph (right)

Monthly average daily minimum temperature **Monthly average daily maximum temperature**

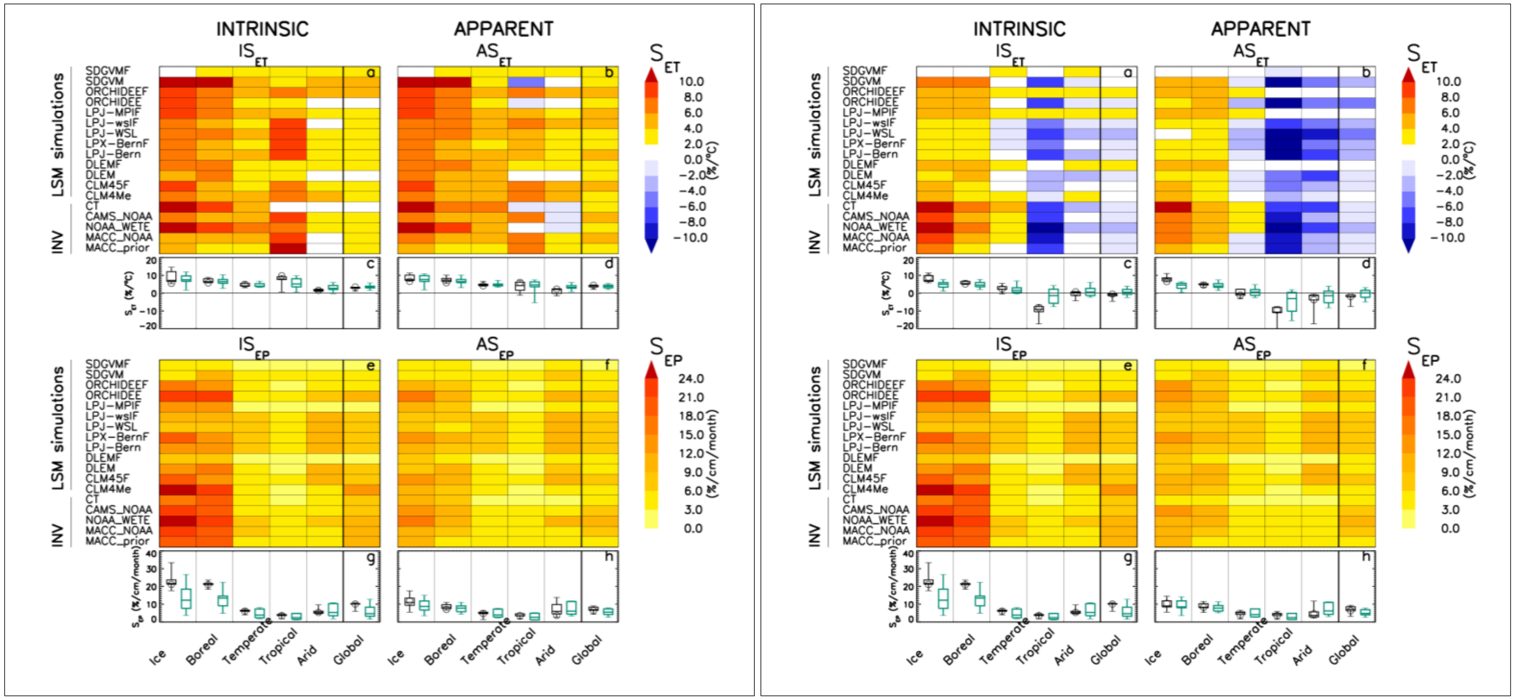

Figure S6: Sensitivity of wetland CH_4 emissions to temperature T and precipitiation P for the five climate zones and at global scale. We use as temperature the monthly average daily minimum (left) and the monthly average daily maximum (right). The emissions are derived from both atmospheric inversions (INV) and land surface model (LSM) simulations. T and P data are from CRU database. The sensitivity of the emissions to T (S_{ET};top) and to P (S_{EP};bottom) are shown. Results for the intrinsic (IS_{ET} and IS_{EP}; left) and for the apparent (AS_{ET} and AS_{EP}; right) sensitivities are shown, respectively. For AS_{ET} and AS_{EP}, the covariance between climate drivers was estimated using the 3-monthly average slopes between P and T. The Whisker plots shown at the bottom of each sensitivy matrix graph are computed from the ensemble of all the inversions (black; and for all the LSMs (green)). The whisker plots show the minimum and maximum S_{ET} or S_{EP} values (bars), and the 25% and 75 % percentiles (boxes). The median values are shown by horizontal line in the box. The values of S_{ET} and S_{EP} obtained from the MACC inversion estimates (MACC_NOAA: the reference inversion in this study) are shown by open circles.

S6: Evolution of the slope between P and T under climate change scenarios

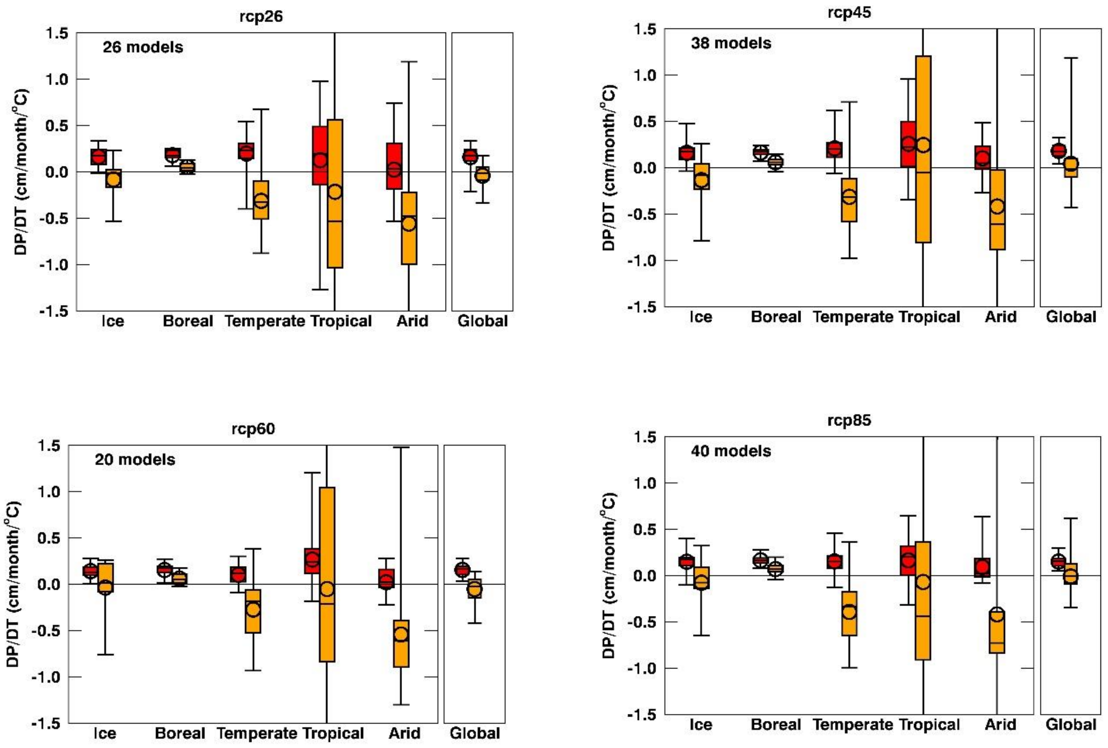

Figure S7: Evolution of the slope between P and T (here DP/DT) based on the projections of an esemble of CMIP5 models. The Whisker plots for DP/DT computed at decadal (red) and inter-annual (orange) scales and for each RCP as function of the five climate zones and at global scale are shown. The Whisker plots show the minimum and maximum of DP/DT values (bars), and the 25% and 75 % percentiles (boxes). The median values are shown by horizontal line in the box. The mean values are shown by open circles. The number of CMIP5 models used for each RCP is indicated in the relevant graph.

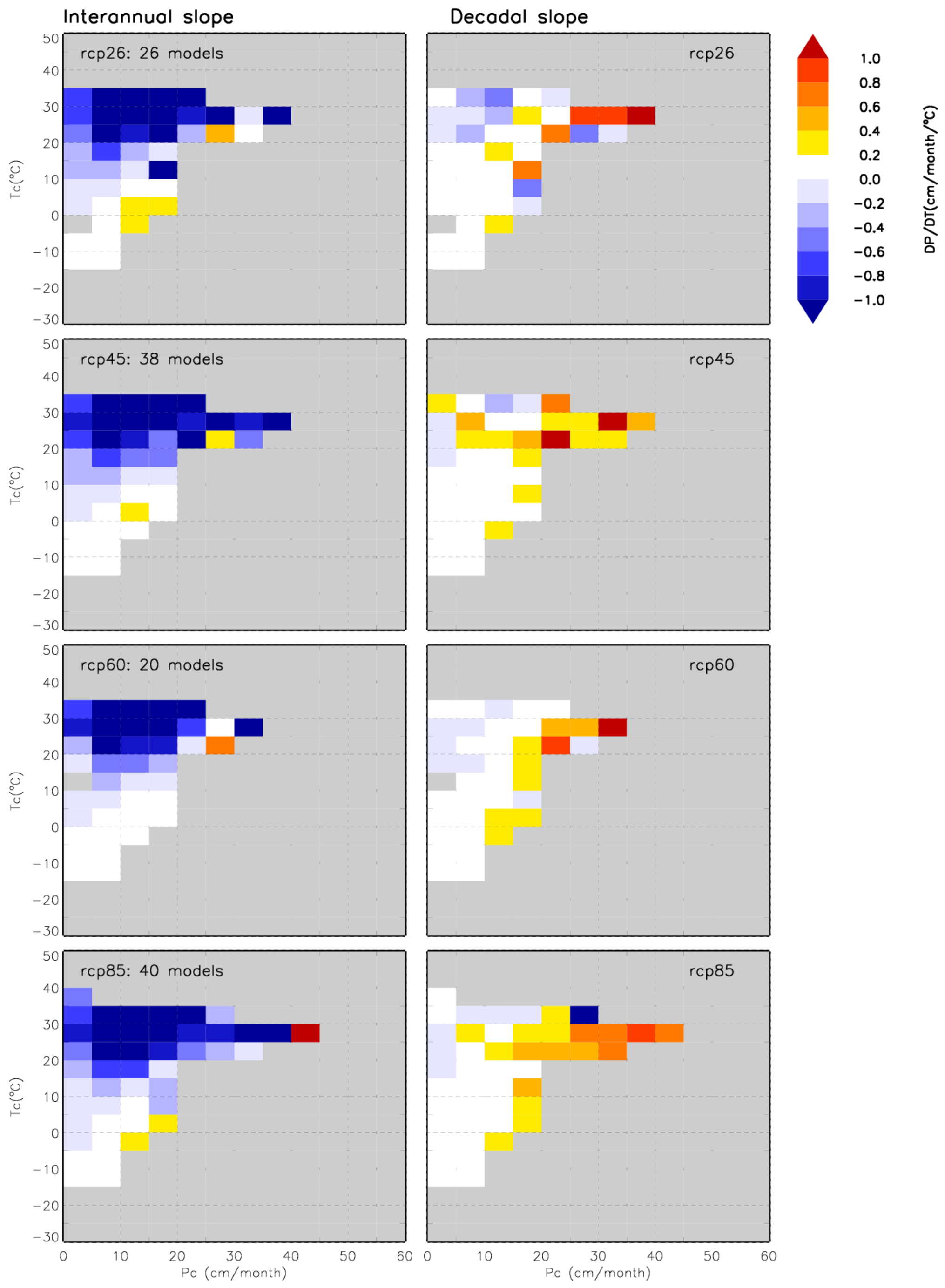

Figure S8: Mean values of the inter-annual (left) and decadal (right) slopes between P and T (DP/DT) derived from the projections of an ensemble models of CMIP5 project as function of temperature (T_c) and precipitation (P_c) classes are shown. The bins (P_c,T_c) are defined by these amplitudes: ΔT_c = 5 ^{o}C and ΔP_c = 5 cm/month. The number of models used for each RCP is indicated in the relevant graph (left hand side)

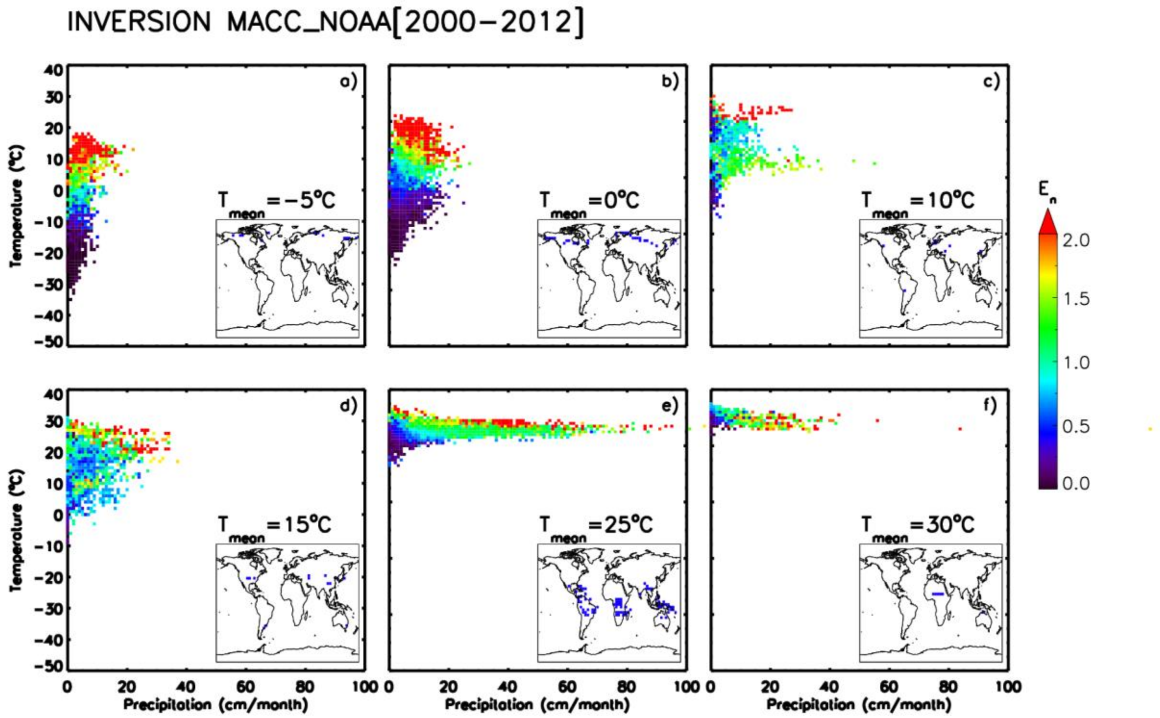

Figure S9: Examples of the response functions of wetland CH_4 emissions to precipitation P and temperature T for pixels having the same mean temperature T_{mean} over 2000-2012 period. Median values of the normalized emission E_n over as function of P and T are shown. The acronyms and period of the emissions are indicated on the top. P and T are from the CRU database. The emissions are from MACC inversions. The map representing the pixels having the same T_{mean} is displayed. The values of T_{mean} are shown on the top of each map. The bin sizes of the classes T_c and P_c are set to 1°C and 1 cm/month, respectively. Only pixels showing wetland emissions are considered.

S7: Observation-driven model simulations of wetland CH_4 emissions using CMIP5 climate projections

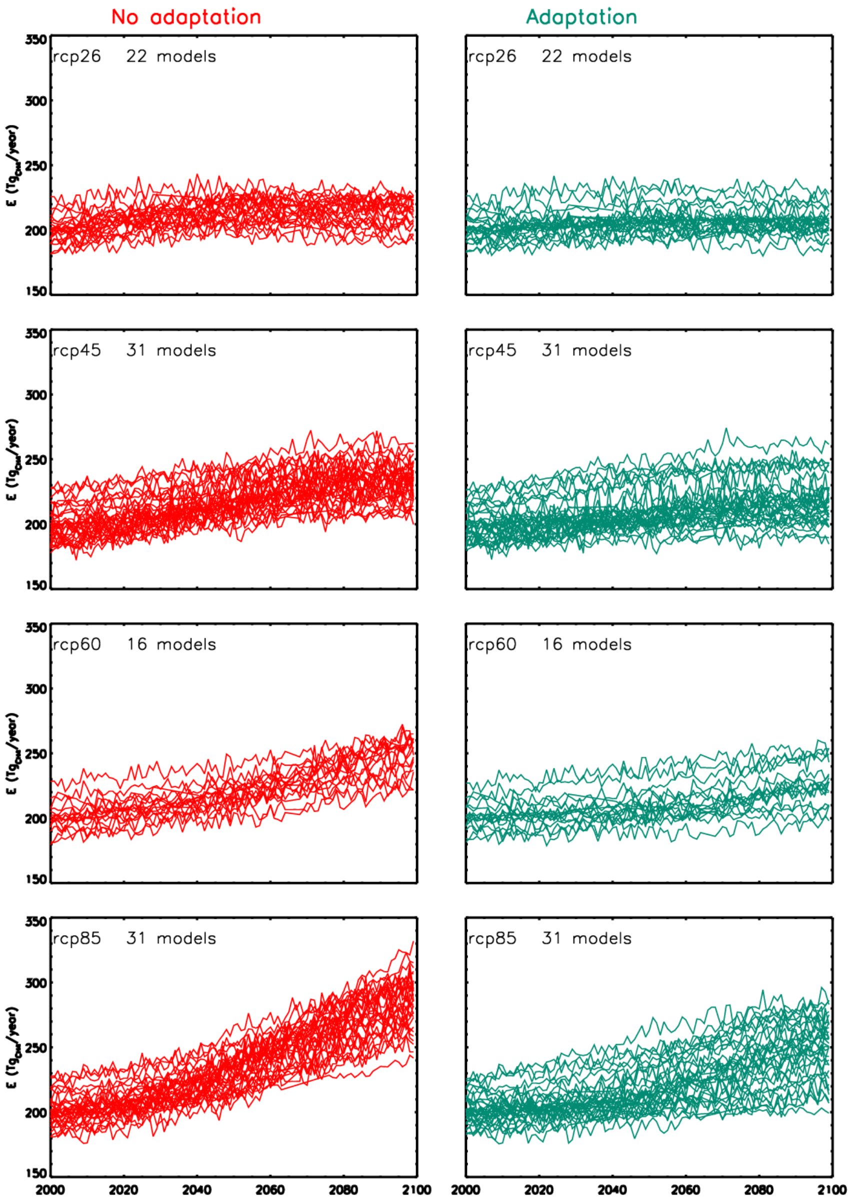

Figure S10: Time series of the computed wetland CH_4 emissions using the observation-driven model and an ensemble of CMIP5 projections (**Methods 4**). The observation-driven method uses for wetland emissions, MACC inversion (MACC_NOAA) and for climate data the temperature and precipitation from CRU. Results obtained for no adaptation (left; red) and a full adaptation (right; green) of wetland to changing climate are shown. The wetland area is considered constant during the simulation period.

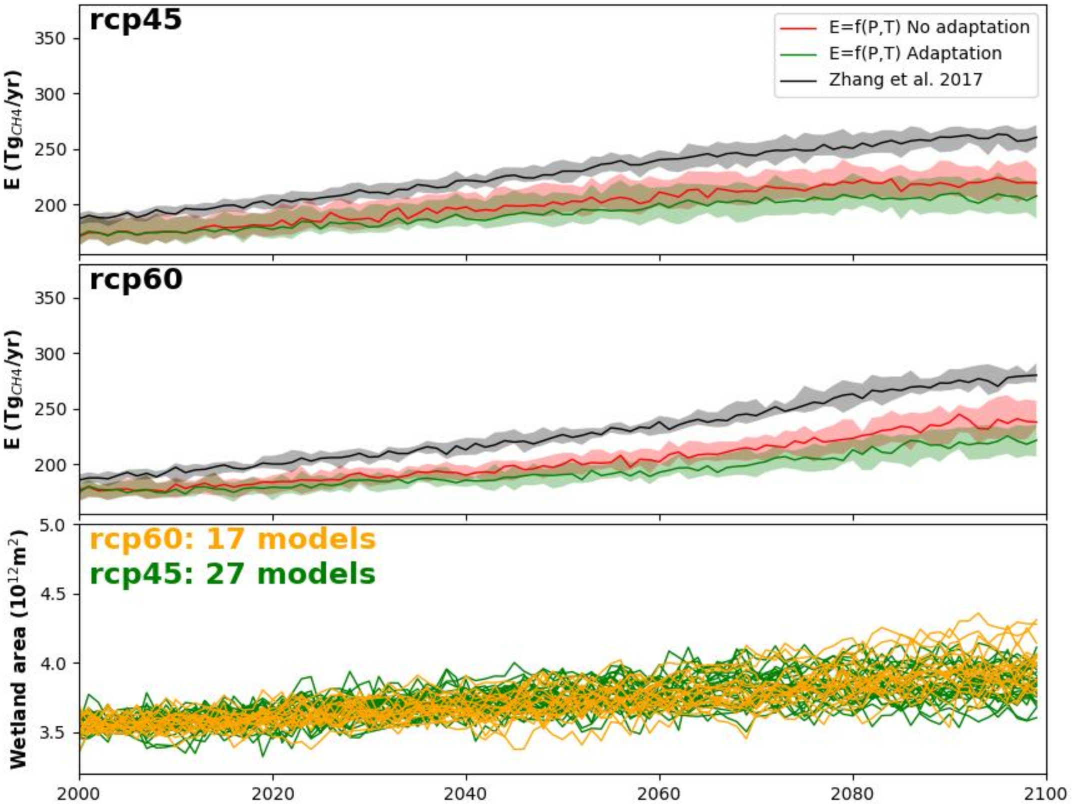

Figure S11: Simulations of wetland CH_4 emissions using the observation-driven model and climate projections of an ensemble of CMIP5 models for the scenarios RCP4.5 (top graph) and RCP6.0 (middle). Two versions of the observation-driven model are considered: simulations without any adaptation of the wetland to the new climate (No Adaptation; red) and simulations with full adapation to climate (Adaptation; green). Results are compared to those of Zhang, *et al.* (19) (grey). The observation-driven model estimates are scaled up by the ratio between the projected wetland areas and the computed areas in year 2000 based on Zhang, *et al.* (19) simulations. Envelopes in the upper and middle panels are enclosed by 25% and 75% percentiles of the ensemble predictions. Solid lines are the median values. The bottom graph shows the yearly variations of the computed wetland areas from Zhang *et al.* (19) from the ensemble of CMIP5 climate models. The number of models used for each RCP is shown in the bottom graph. The observation-driven method uses for wetland methane emissions the MACC inversion (MACC_NOAA).

Table 1: Atmospheric CH_4 inversion products used in this study. Except CarbonTracker-CH_4 (which is based on an Ensemble Kalman Filter system), all inversion products are generated from the TM5-4DVAR system. The surface observations of CH_4 concentrations of NOAA (National Oceanic and Atmospheric Administration) global surface network are used in all the inversion systems. In addition, GOSAT (Greenhouse gas Observing Satellite) retrievals of XCH_4 (from different proxy and full physics retrieval products) are also used simultaneously with the NOAA data in the TM5-4DVAR system.)). The index (C) indicates that two analyses are produced based on two different convection schemes with TM5-4DVAR system. . The CAMS_NOAA is the version "v16r1" of CAMS products.

**Acronym of the inversions**	**Period used in this study**	**Lat x lon (deg.)**	**Observations used in the optimization process**	**Version of GOSAT data**	**Prior wetland emissions**	**References**
					wetland inventory J. Kaplan (2)	Bergamaschi, *et al.* (*2*) http://apps.ecmwf.int/datasets/data/macc-ghg-inversions/
MACC_NOAA	2000-2012	6 x 4	NOAA data			
OCFPv10(C)	2010-2014	6 x 4	NOAA+GOSAT	OCFPv10		
OCFPv60(C)	2010-2014	6 x 4	NOAA+GOSAT	OCFPv60		
FPv237(C)	2010-2014	6 x 4	NOAA+GOSAT	FPv237		
PRv237(C)	2010-2014	6 x 4	NOAA+GOSAT	PRv237		
S2-NOAA	2010-2014	6 x 4	NOAA		LPJ-WHyMe (7, 59)	
S3-NOAA	2010-2014	6 x 4	NOAA		wetland inventory J. Kaplan (2)	
NOAA_WETE	2010-2015	3 x 2	NOAA data		Mean values of seven wetland inventories from the WETCHIMP (7)	
GOSAT_WETE	2010-2015	3 x 2	NOAA+GOSAT	SRPR238		
CAMS_NOAA	2000-2015	3 x 2	NOAA data		wetland inventory J. Kaplan (2)	Segers and Houweling (47) http://apps.ecmwf.int/datasets/data/cams-ghg-inversions/
CarbonTracker-CH_4 (CT)	2000-2010	6 x 4	NOAA data		wetland inventory J. Kaplan (2)	Bruhwiler, *et al.* (49) https://www.esrl.noaa.gov/gmd/ccgg/carbontracker-ch4/

Table 2: Simulations of wetland emissions from land surface models used in this study.

Model	Period	Model references	Project
CLM4Me	1999-2004	Riley, *et al.* (5)	WETCHIMP<br>Melton, *et al.* (7)<br>https://joemelton.weebly.com/wetchimp.html
DLEM		Tian, *et al.* (60)	
LPJ-Bern		Spahni, *et al.* (61)	
LPJ-WSL		Hodson, Poulter, Zimmermann, Prigent and Kaplan (62)	
ORCHIDEE		Ringeval, *et al.* (63)	
SDGVM		Hopcroft, Valdes and Beerling (64)	
CLM4.5F	2000-2012	Riley, *et al.* (5)	Poulter, *et al.* (6)
CTEMF		Riley, *et al.* (5)	
DLEMF		Tian, *et al.* (60)	
JULESF		Hayman, *et al.* (65)	
LPJ-MPIF		Kleinen, Brovkin and Schuldt (66)	
LPJ_WSLF		Hodson, Poulter, Zimmermann, Prigent and Kaplan (62)	
LPX-BernF		Spahni, *et al.* (61)	
ORCHIDEEF		Ringeval, *et al.* (63)	
SDGVMF		Hopcroft, Valdes and Beerling (64)	
TRIPLEXF		Zhu, *et al.* (67)	
VISITF		Ito and Inatomi (68)	

Table 3: Climate data. The different data sets used are built using observations (*in-situ* measurements and satellite) and re-analyses. The soil layers of the ERA-Interim re-analyses are: layer 1 (0-0.07 m), layer 2 (0.0-0.28 m), layer 3 (0.28-1.0 m), and layer 4 (1.0-2.89 m)

**Data base**	**Climate variable**	**Variable Acronym**	**Version of the product used**	**Period used in this study**	**References**
CRU	Daily mean temperature	CRUtmp	CRU TS v4.01	1994-2015	Harris, Jones, Osborn and Lister (31) https://crudata.uea.ac.uk/cru/data/hrg/
	monthly average daily minimum	CRUtmn			
	monthly average daily maximum	CRUtmx			
ERA-Interim	2m air temperature	ECMWFt2m	ERA-Interim	2000-2012	Dee, *et al.* (51) http://apps.ecmwf.int/datasets/data/interim-full-moda/levtype=sfc/;
	Soil temperature, layer 1	ECMWFstl1			
	Soil temperature, layer 2	ECMWFstl2			
	Soil temperature, layer 3	ECMWFstl3			
	Soil temperature, layer 4	ECMWFstl4			
CRU	Precipitation	CRU_P	CRU TS v4.01	2000-2015	Harris, Jones, Osborn and Lister (31)
GPCC	Precipitation	GPCC		2000-2012	Schneider, *et al.* (42) ftp://ftp-anon.dwd.de/pub/data/gpcc/html/download_gate.html
GPCP	Precipitation	GPCP	Monthly mean	2000-2012	Adler, *et al.* (53) https://www.esrl.noaa.gov/psd/data/gridded/data.gpcp.html
ERA-Interim	Water content in the soil, layer 1	ECMWFswvl1	ERA-Interim	2000-2012	Dee, *et al.* (51) http://apps.ecmwf.int/datasets/data/interim-full-moda/levtype=sfc/;
	Water content in the soil, layer 2	ECMWFswvl2			
	Water content in the soil, layer 3	ECMWFswvl3			
	Water content in the soil, layer 4	ECMWFswvl4			
GRACE	Water in the soil	GRACE	DSTvSCS1411	2003-2012	Swenson (54) http://dx.doi.org/10.5067/TELND-NC005
MODIS	Land Surface Water Index (LSWI)	MODIS	MCD43A3	2004-2010	Schaaf and Wang (55) https://modis.gsfc.nasa.gov/data/dataprod/mod43.php

Table 4: CMIP5 projections: List of models used in this study. Cross marks indicate which RCP scenarios are used. Other details can be found from https://cmip.llnl.gov/cmip5/

Model	Country	Institute	Lat x lon (deg.)	RCP			
				2.6	4.5	6.0	8.5
ACCESS1.0	Australia	CSIRO (Commonwealth Scientific and Industrial Research Organisation, Australia), and BOM (Bureau of Meteorology, Australia)	1.25 x1.88		X		X
ACCESS1.3	Australia	CSIRO (Commonwealth Scientific and Industrial Research Organisation, Australia), and BOM (Bureau of Meteorology, Australia)	1.25 x 1.88		X		X
bcc-csm1-1	China	Beijing Climate Center, China Meteorological Administration	2.79 x 2.81	X	X	X	X
Bcc-csm1-1-m	China	Beijing Climate Center, China Meteorological Administration	2.79 x 2.81	X	X	X	X
BNU-ESM	China	College of Global Change and Earth System Science, Beijing Normal University	2.79 x 2.81	X	x		X
CCSM4	United States	National Center for Atmospheric Research	0.94 x 1.25	X	X	X	X
CESM1_CAM5	United States	National Science Foundation, Department of Energy, National Center for Atmospheric Research	0.94 x 1.25	X	X		X
CMCC-CESM	Italy	Centro Euro-Mediterraneo per I Cambiamenti Climatici	3.44 x 3.75				X
CMCC-CM	Italy	Centro Euro-Mediterraneo per I Cambiamenti Climatici	0.75 x 0.75		X		X
CMCC-CMS	Italy	Centro Euro-Mediterraneo per I Cambiamenti Climatici	3.71 x 3.75		X		X
CNRM-CM5	France	Centre National de Recherches Meteorologiques / Centre Europeen de Recherche et Formation Avancees en Calcul Scientifique	1.40 x 1.41		X		X
CSIRO-Mk3.6.0	Australia	CSIRO (Commonwealth Scientific and Industrial Research Organisation, Australia), and BOM (Bureau of Meteorology)	1.87 x 1.87	X	X	X	X
CanESM2	Canada	Canadian Centre for Climate Modelling and Analysis	2.79 x 2.81	X	X		
FGOALS-g2	China	LASG, Institute of Atmosphere Physics, Chinese Academic of Sciences	2.79 x 2.81	X	X		X
FIO-ESM	China	The First Institute of Oceanography, SOA, China		X	X		X
GFDL-CM3	United States	Geophysical Fluid Dynamics Laboratory	2.00 x 2.50		X	X	X
GFDL-ESM2G	United States	Geophysical Fluid Dynamics Laboratory	2.02 x 2.00	X	X	X	X
GFDL-ESM2M	United States	Geophysical Fluid Dynamics Laboratory	2.02 x 2.50	X	X	X	
GISS-E2-H	United States	NASA Goddard Institute for Space Studies	2.00 x 2.50	X	X	X	X
GISS-E2-H-CC	United States	NASA Goddard Institute for Space Studies	2.00 x 2.50		X		
GISS-E2-R	United States	NASA Goddard Institute for Space Studies	2.00 x 2.50	X	X	X	X
GISS-E2-R-CC	Unites States	NASA Goddard Institute for Space Studies	2.00 x 2.50		X		X
HadGEM2-AO	United Kingdom	Met Office Hadley Centre	1.25 x 1.88	X	X	X	X
HadGEM2-CC	United Kingdom	Met Office Hadley Centre	1.25 x 1.88		X		X
HadGEM2-ES	United Kingdom	Met Office Hadley Centre	1.25 x 1.88	X	X	X	X
IPSL-CM5A-LR	France	Institut Pierre-Simon Laplace	1.89 x 3.75	X	X	X	X
IPSL-CM5A-MR	France	Institut Pierre-Simon Laplace	1.27 x 2.50	X	X	X	X
IPSL-CM5B-LR	France	Institut Pierre-Simon Laplace	1.89 x 3.75		X		X
MIROC-ESM	Japan		2.79 x 2.81	X	X	X	X

MIROC-ESM-CHEM	Japan	Japan Agency for Marine-Earth Science and Technology, Atmosphere and Ocean Research Institute (The University of Tokyo), and National Institute for Environmental Studies	2.79 x 2.81	X	X	X	X
MIROC5	Japan	Japan Agency for Marine-Earth Science and Technology, Atmosphere and Ocean Research Institute (The University of Tokyo), and National Institute for Environmental Studies	1.40 x 1.41	X	X		X
MPI-ESM-LR	Germany	Max Planck Institute for Meteorology (MPI-M)	1.87 x 1.88	X	X		X
MPI-ESM-MR	Germany	Max Planck Institute for Meteorology (MPI-M)	1.87 x 1.88	X	X		X
MRI-CGCM3	Japan	Meteorological Research Institute	1.12 x 1.13	X	X	X	X
MRI-ESM1	Japan	Meteorological Research Institute	1.12 x 1.13	X	X		X
NorESM1-M	Noarway	Norwegian Climate Centre	1.89 x 2.50	X	X	X	X
NorESM1-ME	Norway	Norwegian Climate Centre	1.89 x2.50	X	X	X	X
Inmcm4	Russia	Institute of Numerical Mathematics	1.50 x 2.00		X		X

REFERENCES AND NOTES

1. IPCC, in *Climate Change 2013 The Physical Science Basis. Contribution of Working Group I to the Fifth Assessment Report of the Intergovernmental Panel on Climate Change*, T. F. Stocker, D. Qin, G.-K. Plattner, M. Tignor, S. K. Allen, J. Boschung, A. Nauels, Y. Xia, V. Bex, P. M. Midgley, Eds. (Cambridge Univ. Press, 2013). Chapter 6: Carbon and Other Biogeochemical Cycles. pp. 466–570.

2. P. Bergamaschi, S. Houweling, A. Segers, M. Krol, C. Frankenberg, R. A. Scheepmaker, E. Dlugokencky, S. C. Wofsy, E. A. Kort, C. Sweeney, T. Schuck, C. Brenninkmeijer, H. Chen, V. Beck, C. Gerbig, Atmospheric CH_4 in the first decade of the 21st century: Inverse modeling analysis using SCIAMACHY satellite retrievals and NOAA surface measurements. *J. Geophys. Res. Atmos.* **118**, 7350–7369 (2013).

3. R. Wania, I. Ross, I. C. Prentice, Implementation and evaluation of a new methane model within a dynamic global vegetation model: LPJ-WHyMe v1.3.1. *Geosci. Model Dev.* **3**, 565–584 (2010).

4. M. Saunois, P. Bousquet, B. Poulter, A. Peregon, P. Ciais, J. G. Canadell, E. J. Dlugokencky, G. Etiope, D. Bastviken, S. Houweling, G. Janssens-Maenhout, F. N. Tubiello, S. Castaldi, R. B. Jackson, M. Alexe, V. K. Arora, D. J. Beerling, P. Bergamaschi, D. R. Blake, G. Brailsford, V. Brovkin, L. Bruhwiler, C. Crevoisier, P. Crill, K. Covey, C. Curry, C. Frankenberg, N. Gedney, L. Höglund-Isaksson, M. Ishizawa, A. Ito, F. Joos, H. S. Kim, T. Kleinen, P. Krummel, J.-F. Lamarque, R. Langenfelds, R. Locatelli, T. Machida, S. Maksyutov, K. C. McDonald, J. Marshall, J. R. Melton, I. Morino, V. Naik, S. O'Doherty, F.-J. W. Parmentier, P. K. Patra, C. Peng, S. Peng, G. P. Peters, I. Pison, C. Prigent, R. Prinn, M. Ramonet, W. J. Riley, M. Saito, M. Santini, R. Schroeder, I. J. Simpson, R. Spahni, P. Steele, A. Takizawa, B. F. Thornton, H. Tian, Y. Tohjima, N. Viovy, A. Voulgarakis, M. van Weele, G. R. van der Werf, R. Weiss, C. Wiedinmyer, D. J. Wilton, A. Wiltshire, D. Worthy, D. Wunch, X. Xu, Y. Yoshida, B. Zhang, Z. Zhang, Q. Zhu, The global methane budget 2000-2012. *Earth Syst. Sci. Data* **8**, 697–751 (2016).

5. W. J. Riley, Z. M. Subin, D. M. Lawrence, S. C. Swenson, M. S. Torn, L. Meng, N. M. Mahowald, P. Hess, Barriers to predicting changes in global terrestrial methane fluxes: Analyses using CLM4Me, a methane biogeochemistry model integrated in CESM. *Biogeosciences* **8**, 1925–1953 (2011).

6. P. Poulter, P. Bousquet, J. G. Canadell, P. Ciais, A. Peregon, M. Saunois, V. K. Arora, D. J. Beerling, V. Brovkin, C. D. Jones, F. Joos, N. Gedney, A. Ito, T. Kleinen, C. D. Koven, K. M. Donald, J. R. Melton, C. Peng, S. Peng, C. Prigent, R. Schroeder, W. J. Riley, M. Saito, R. Spahni, H. Tian, L. Taylor, N. Viovy, D. Wilton, A. Wiltshire, X. Xu, B. Zhang, Z. Zhang, Q. Zhu, Global wetland contribution to 2000–2012 atmospheric methane growth rate dynamics. *Environ. Res. Lett.* **12**, 094013 (2017).

7. J. R. Melton, R. Wania, E. L. Hodson, B. Poulter, B. Ringeval, R. Spahni, T. Bohn, C. A. Avis, D. J. Beerling, G. Chen, A. V. Eliseev, S. N. Denisov, P. O. Hopcroft, D. P. Lettenmaier, W. J. Riley, J. S. Singarayer, Z. M. Subin, H. Tian, S. Zürcher, V. Brovkin, P. M. van Bodegom, T. Kleinen, Z. C. Yu, J. O. Kaplan, Present state of global wetland extent and wetland methane modelling: Conclusions from a model inter-comparison project (WETCHIMP). *Biogeosciences* **10**, 753–788 (2013).

8. S. Kirschke, P. Bousquet, P. Ciais, M. Saunois, J. G. Canadell, E. J. Dlugokencky, P. Bergamaschi, D. Bergmann, D. R. Blake, L. Bruhwiler, P. Cameron-Smith, S. Castaldi, F. Chevallier, L. Feng, A. Fraser, M. Heimann, E. L. Hodson, S. Houweling, B. Josse, P. J. Fraser, P. B. Krummel, J. F. Lamarque, R. L. Langenfelds, C. le Quéré, V. Naik, S. O'Doherty, P. I. Palmer, I. Pison, D. Plummer, B. Poulter, R. G. Prinn, M. Rigby, B. Ringeval, M. Santini, M. Schmidt, D. T. Shindell, I. J. Simpson, R. Spahni, L. P. Steele, S. A. Strode, K. Sudo, S. Szopa, G. R. van der Werf, A. Voulgarakis, M. van Weele, R. F. Weiss, J. E. Williams, G. Zeng, Three decades of global methane sources and sinks. *Nat. Geosci.* 6, 813–823 (2013).

9. M. Köchy, R. Hiederer, A. Freibauer, Global distribution of soil organic carbon – Part 1: Masses and frequency distributions of SOC stocks for the tropics, permafrost regions, wetlands, and the world. *SOIL* **1**, 351–365 (2015).

10. E. Gorham, Northern peatlands: Role in the carbon cycle and probable responses to climatic warming. *Ecol. Appl.* **1**, 182–195 (1991).

11. F. M. O’Connor, O. Boucher, N. Gedney, C. D. Jones, G. A. Folberth, R. Coppell, P. Friedlingstein, W. J. Collins, J. Chappellaz, J. Ridley, C. E. Johnson, Possible role of wetlands, permafrost, and methane hydrates in the methane cycle under future climate change: A review. *Rev. Geophys.* **48**, RG4005 (2010).

12. T. R. Moore, N. T. Roulet, J. M. Waddington, Uncertainty in predicting the effect of climatic change on the carbon cycling of canadian peatlands. *Clim. Change* **40**, 229–245 (1998).

13. T. R. Christensen, A. Ekberg, L. Ström, M. Mastepanov, N. Panikov, M. Öquist, B. H. Svensson, H. Nykänen, P. J. Martikainen, H. Oskarsson, Factors controlling large scale variations in methane emissions from wetlands. *Geophys. Res. Lett.* **30**, 1414 (2003).

14. A. Arneth, S. Sitch, A. Bondeau, K. Butterbach-Bahl, P. Foster, N. Gedney, N. de Noblet-Ducoudré, I. C. Prentice, M. Sanderson, K. Thonicke, R. Wania, S. Zaehle, From biota to chemistry and climate: Towards a comprehensive description of trace gas exchange between the biosphere and atmosphere. *Biogeosciences* **7**, 121–149 (2010).

15. V. Jerman, M. Metje, I. Mandić-Mulec, P. Frenzel, Wetland restoration and methanogenesis: The activity of microbial populations and competition for substrates at different temperatures. *Biogeosciences* **6**, 1127–1138 (2009).

16. K. E. Trenberth, A. Dai, R. M. Rasmussen, D. B. Parsons, The changing character of precipitation. *Bull. Am. Meteorol. Soc.* **84**, 1205–1218 (2003).

17. A. E. Putnam, W. S. Broecker, Human-induced changes in the distribution of rainfall. *Sci. Adv.* **3**, e1600871 (2017).

18. R. Alkama, Human influence on changes in the distribution of land precipitation. *J. Hydrol.* **511**, 589–593 (2014).

19. Z. Zhang, N. E. Zimmermann, A. Stenke, X. Li, E. L. Hodson, G. Zhu, C. Huang, B. Poulter, Emerging role of wetland methane emissions in driving 21st century climate change. *Proc. Natl. Acad. Sci. U.S.A.* **114**, 9647–9652 (2017).

20. J. E. Campbell, J. A. Berry, U. Seibt, S. J. Smith, S. A. Montzka, T. Launois, S. Belviso, L. Bopp, M. Laine, Large historical growth in global terrestrial gross primary production. *Nature* **544**, 84–87 (2017).

21. E. A. G. Schuur, J. Bockheim, J. G. Canadell, E. Euskirchen, C. B. Field, S. V. Goryachkin, S. Hagemann, P. Kuhry, P. M. Lafleur, H. Lee, G. Mazhitova, F. E. Nelson, A. Rinke, V. E. Romanovsky, N. Shiklomanov, C. Tarnocai, S. Venevsky, J. G. Vogel, S. A. Zimov, Vulnerability of permafrost carbon to climate change: Implications for the global carbon cycle. *Bioscience* **58**, 701–714 (2008).

22. A. M. R. Petrescu, A. Lohila, J.-P. Tuovinen, D. D. Baldocchi, A. R. Desai, N. T. Roulet, T. Vesala, A. J. Dolman, W. C. Oechel, B. Marcolla, T. Friborg, J. Rinne, J. H. Matthes, L. Merbold, A. Meijide, G. Kiely, M. Sottocornola, T. Sachs, D. Zona, A. Varlagin, D. Y. F. Lai, E. Veenendaal, F.-J. W. Parmentier, U. Skiba, M. Lund, A. Hensen, J. van Huissteden, L. B. Flanagan, N. J. Shurpali, T. Grünwald, E. R. Humphreys, M. Jackowicz-Korczyński, M. A. Aurela, T. Laurila, C. Grüning, C. A. R. Corradi, A. P. Schrier-Uijl, T. R. Christensen, M. P. Tamstorf, M. Mastepanov, P. J. Martikainen, S. B. Verma, C. Bernhofer, A. Cescatti, The uncertain climate footprint of wetlands under human pressure. *Proc. Natl. Acad. Sci. U.S.A.* **112**, 4594–4599 (2015).

23. E. M. Volodin, Methane cycle in the INM RAS climate model. *Izv. Atmos. Ocean. Phys.* **44**, 153–159 (2008).

24. B. D. Stocker, R. Roth, F. Joos, R. Spahni, M. Steinacher, S. Zaehle, L. Bouwman, Xu-Ri, I. C. Prentice, Multiple greenhouse-gas feedbacks from the land biosphere under future climate change scenarios. *Nat. Clim. Chang.* **3**, 666–672 (2013), 672.

25. D. T. Shindell, B. P. Walter, G. Faluvegi, Impacts of climate change on methane emissions from wetlands. *Geophys. Res. Lett.* **31**, L21202 (2004).

26. B. Ringeval, P. Friedlingstein, C. Koven, P. Ciais, N. de Noblet-Ducoudré, B. Decharme, P. Cadule, Climate-CH_4 feedback from wetlands and its interaction with the climate-CO_2 feedback. *Biogeosciences* **8**, 2137–2157 (2011).

27. N. Gedney, P. M. Cox, C. Huntingford, Climate feedback from wetland methane emissions. *Geophys. Res. Lett.* **31**, L20503 (2004).

28. S. Houweling, P. Bergamaschi, F. Chevallier, M. Heimann, T. Kaminski, M. Krol, A. M. Michalak, P. Patra, Global inverse modeling of CH_4 sources and sinks: An overview of methods. *Atmos. Chem. Phys.* **17**, 235–256 (2017).

29. S. Pandey, S. Houweling, M. Krol, I. Aben, G. Monteil, N. Nechita-Banda, E. J. Dlugokencky, R. Detmers, O. Hasekamp, X. Xu, W. J. Riley, B. Poulter, Z. Zhang, K. C. McDonald, J. W. C. White, P. Bousquet, T. Röckmann, Enhanced methane emissions from tropical wetlands during the 2011 La Niña. *Sci. Rep.* **7**, 45759 (2017).

30. A. A. Bloom, P. I. Palmer, A. Fraser, D. S. Reay, C. Frankenberg, Large-scale controls of methanogenesis inferred from methane and gravity spaceborne data. *Science* **327**, 322–325 (2010).

31. I. Harris, P. D. Jones, T. J. Osborn, D. H. Lister, Updated high-resolution grids of monthly climatic observations – The CRU TS3.10 dataset. *Int. J. Climatol.* **34**, 623–642 (2014).

32. K. E. Trenberth, D. J. Shea, Relationships between precipitation and surface temperature. *Geophys. Res. Lett.* **32**, L14703 (2005).

33. M. Jung, M. Reichstein, C. R. Schwalm, C. Huntingford, S. Sitch, A. Ahlström, A. Arneth, G. Camps-Valls, P. Ciais, P. Friedlingstein, F. Gans, K. Ichii, A. K. Jain, E. Kato, D. Papale, B. Poulter, B. Raduly, C. Rödenbeck, G. Tramontana, N. Viovy, Y. P. Wang, U. Weber, S. Zaehle, N. Zeng, Compensatory water effects link yearly global land CO_2 sink changes to temperature. *Nature* **541**, 516 (2017), 520.

34. K. E. Taylor, R. J. Stouffer, G. A. Meehl, An overview of CMIP5 and the experiment design. *Bull. Am. Meteorol. Soc.* **93**, 485–498 (2012).

35. S. Saarnio, S. Järviö, T. Saarinen, H. Vasander, J. Silvola, Minor changes in vegetation and carbon gas balance in a boreal mire under a raised CO_2 or NH_4NO_3 supply. *Ecosystems* **6**, 0046–0060 (2003).

36. J. P. Megonigal, W. Schlesinger, Enhanced CH_4 emissionsfrom a wetland soil exposed to elevated CO_2. *Biogeochemistry* **37**, 77–88 (1997).

37. P. R. Hutchin, M. C. Press, J. A. Lee, T. W. Ashenden, Elevated concentrations of CO_2 may double methane emissions from mires. *Glob. Chang. Biol.* 1, 125–128 (1995).

38. D. T. Shindell, O. Pechony, A. Voulgarakis, G. Faluvegi, L. Nazarenko, J. F. Lamarque, K. Bowman, G. Milly, B. Kovari, R. Ruedy, G. A. Schmidt, Interactive ozone and methane chemistry in GISS-E2 historical and future climate simulations. *Atmos. Chem. Phys.* **13**, 2653–2689 (2013).

39. B. Zhang, H. Tian, C. Lu, G. Chen, S. Pan, C. Anderson, B. Poulter, Methane emissions from global wetlands: An assessment of the uncertainty associated with various wetland extent data sets. *Atmos. Environ.* **165**, 310–321 (2017).

40. D. T. Shindell, J.-F. Lamarque, M. Schulz, M. Flanner, C. Jiao, M. Chin, P. J. Young, Y. H. Lee, L. Rotstayn, N. Mahowald, G. Milly, G. Faluvegi, Y. Balkanski, W. J. Collins, A. J. Conley, S. Dalsoren, R. Easter, S. Ghan, L. Horowitz, X. Liu, G. Myhre, T. Nagashima, V. Naik, S. T. Rumbold, R. Skeie, K. Sudo, S. Szopa, T. Takemura, A. Voulgarakis, J.-H. Yoon, F. Lo, Radiative forcing in the ACCMIP historical and future climate simulations. *Atmos. Chem. Phys.* **13**, 2939–2974 (2013).

41. B. Lehner, P. Dölla, Development and validation of a global database of lakes, reservoirs and wetlands. *J. Hydrol.* **296**, 1–22 (2004).

42. U. Schneider, A. Becker, P. Finger, A. Meyer-Christoffer, M. Ziese, B. Rudolf, GPCC's new land surface precipitation climatology based on quality-controlled in situ data and its role in quantifying the global water cycle. *Theor. Appl. Climatol.* **115**, 15–40 (2014).

43. M. Kottek, J. Grieser, C. Beck, B. Rudolf, F. Rubel, World map of the Köppen-Geiger climate classification updated. *Meteorol. Z.* **15**, 259–263 (2006).

44. J. F. Meirink, P. Bergamaschi, C. Frankenberg, Monica T. S. d'Amelio, E. J. Dlugokencky, L. V. Gatti, S. Houweling, J. B. Miller, T. Röckmann, M. Gabriella Villani, M. C. Krol, Four-dimensional variational data assimilation for inverse modelling of atmospheric methane emissions: Analysis of SCIAMACHY observations. *J. Geophys. Res.* **113**, D17301 (2008).

45. M. Krol, S. Houweling, B. Bregman, M. van den Broek, A. Segers, P. van Velthoven, W. Peters, F. Dentener, P. Bergamaschi, The two-way nested global chemistry-transport zoom model TM5: Algorithm and applications. *Atmos. Chem. Phys.* **5**, 417–432 (2005).

46. J. C. Gilbert, C. Lemaréchal, Some numerical experiments with variable-storage quasi-Newton algorithms. *Math. Program.* **45**, 407–435 (1989).

47. A. Segers, S. Houweling, "Description of the CH_4 inversion production chain" (Copernicus Atmosphere Monitoring Service, 2017).

48. E. N. Koffi, P. Bergamaschi, “Evaluation of Copernicus Atmosphere Monitoring Service methane products” (JRC Technical Reports, Joint Research Centre, 2018).

49. L. Bruhwiler, E. Dlugokencky, K. Masarie, M. Ishizawa, A. Andrews, J. Miller, C. Sweeney, P. Tans, D. Worthy, CarbonTracker-CH_4: An assimilation system for estimating emissions of atmospheric methane. *Atmos. Chem. Phys.* 14, 8269–8293 (2014).

50. J. O. Kaplan, Wetlands at the Last Glacial Maximum: Distribution and methane emissions. *Geophys. Res. Lett.* **29**, 3-1–3-4 (2002).

51. D. P. Dee, S. M. Uppala, A. J. Simmons, P. Berrisford, P. Poli, S. Kobayashi, U. Andrae, M. A. Balmaseda, G. Balsamo, P. Bauer, P. Bechtold, A. C. M. Beljaars, L. van de Berg, J. Bidlot, N. Bormann, C. Delsol, R. Dragani, M. Fuentes, A. J. Geer, L. Haimberger, S. B. Healy, H. Hersbach, E. V. Hólm, L. Isaksen, P. Kållberg, M. Köhler, M. Matricardi, A. P. McNally, B. M. Monge-Sanz, J.-J. Morcrette, B. K. Park, C. Peubey, P. de Rosnay, C. Tavolato, J.-N. Thépaut, F. Vitart, The ERA-Interim reanalysis: Configuration and performance of the data assimilation system. *Q. J. Roy. Meteorol. Soc.* **137**, 553–597 (2011).

52. U. Schneider, A. Becker, P. Finger, A. Meyer-Christoffer, M. Ziese, “GPCC Full Data Monthly Product Version 2018 at 0.5°: Monthly land-surface precipitation from rain-gauges built on GTS-based and historical data” (Global Precipitation Climatology Centre, 2018).

53. R. F. Adler, G. J. Huffman, A. Chang, R. Ferraro, P. P. Xie, J. Janowiak, B. Rudolf, U. Schneider, S. Curtis, D. Bolvin, A. Gruber, J. Susskind, P. Arkin, E. Nelkin, The Version-2 Global Precipitation Climatology Project (GPCP) monthly precipitation analysis (1979–present). *J. Hydrometeorol.* **4**, 1147–1167 (2003).

54. S. C. Swenson, “GRACE monthly land water mass grids NETCDF RELEASE 5.0. Ver. 5.0” (PO.DAAC, 2012).

55. Z. Wang, C. Schaaf, “MCD43A1 MODIS/Terra+Aqua BRDF/Albedo model parameters daily L3 global - 500m V006” (NASA EOSDIS Land Processes DAAC, 2015).

56. K. Chandrasekar, M. V. R. Sesha Sai, P. S. Roy, R. S. Dwevedi, Land Surface Water Index (LSWI) response to rainfall and NDVI using the MODIS Vegetation Index product. *Int. J. Remote Sens.* **31**, 3987–4005 (2010).

57. IPCC, in *Climate Change 2014: Synthesis Report. Contribution of Working Groups I, II and III to the Fifth Assessment Report of the Intergovernmental Panel on Climate Change*, Core Writing Team, R. K. Pachauri, L. A. Meyer, Eds. (IPCC, 2014), chapters 2–4, pp. 56–112.

58. D. Shepard, A two-dimensional interpolation function for irregularly-spaced data, in *Proceedings of the 1968 23rd ACM National Conference*, (ACM, 1968), pp 517–524.

59. R. Wania, J. R. Melton, E. L. Hodson, B. Poulter, B. Ringeval, R. Spahni, T. Bohn, C. A. Avis, G. Chen, A. V. Eliseev, P. O. Hopcroft, W. J. Riley, Z. M. Subin, H. Tian, P. M. van Bodegom, T. Kleinen, Z. C. Yu, J. S. Singarayer, S. Zürcher, D. P. Lettenmaier, D. J. Beerling, S. N. Denisov, C. Prigent, F. Papa, J. O. Kaplan, Present state of global wetland extent and wetland methane modelling: Methodology of a model inter-comparison project (WETCHIMP). *Geosci. Model Dev.* **6**, 617–641 (2013).

60. H. Tian, G. Chen, M. Liu, C. Zhang, G. Sun, C. Lu, X. Xu, W. Ren, S. Pan, A. Chappelka, Model estimates of net primary productivity, evapotranspiration, and water use efficiency in the terrestrial ecosystems of the southern United States during 1895–2007. *For. Ecol. Manage.* **259**, 1311–1327 (2010).

61. R. Spahni, R. Wania, L. Neef, M. van Weele, I. Pison, P. Bousquet, C. Frankenberg, P. N. Foster, F. Joos, I. C. Prentice, P. van Velthoven, Constraining global methane emissions and uptake by ecosystems. *Biogeosciences* **8**, 1643–1665 (2011).

62. E. L. Hodson, B. Poulter, N. E. Zimmermann, C. Prigent, J. O. Kaplan, The El Niño–Southern Oscillation and wetland methane interannual variability. *Geophys. Res. Lett.* **38**, L08810 (2011).

63. B. Ringeval, N. de Noblet-Ducoudré, P. Ciais, P. Bousquet, C. Prigent, F. Papa, W. B. Rossow, An attempt to quantify the impact of changes in wetland extent on methane emissions on the seasonal and interannual time scales. *Global Biogeochem. Cycles* **24**, GB2003 (2010).

64. P. O. Hopcroft, P. J. Valdes, D. J. Beerling, Simulating idealized Dansgaard-Oeschger events and their potential impacts on the global methane cycle. *Quat. Sci. Rev.* **30**, 3258–3268 (2011).

65. G. D. Hayman, F. M. O'Connor, M. Dalvi, D. B. Clark, N. Gedney, C. Huntingford, C. Prigent, M. Buchwitz, O. Schneising, J. P. Burrows, C. Wilson, N. Richards, M. Chipperfield, Comparison of the HadGEM2 climate-chemistry model against in situ and SCIAMACHY atmospheric methane data. *Atmos. Chem. Phys.* **14**, 13257–13280 (2014).

66. T. Kleinen, V. Brovkin, R. J. Schuldt, A dynamic model of wetland extent and peat accumulation: Results for the Holocene. *Biogeosciences* **9**, 235–248 (2012).

67. Q. Zhu, C. Peng, H. Chen, X. Fang, J. Liu, H. Jiang, Y. Yang, G. Yang, Estimating global natural wetland methane emissions using process modelling: Spatio-temporal patterns and contributions to atmospheric methane fluctuations. *Glob. Ecol. Biogeogr.* **24**, 959–972 (2015).

68. A. Ito, M. Inatomi, Use of a process-based model for assessing the methane budgets of global terrestrial ecosystems and evaluation of uncertainty. *Biogeosciences* **9**, 759–773 (2012).
